# Mechanistic Model for the Hsp90-Driven Opening of Human Argonaute

**DOI:** 10.1021/acs.jcim.0c00053

**Published:** 2020-02-25

**Authors:** Silvia Rinaldi, Giorgio Colombo, Antonella Paladino

**Affiliations:** † Istituto di Science e Tecnologie Chimiche “Giulio Natta” SCITEC, CNR, via Mario Bianco 9, 20131, Milan, Italy; ‡ Dipartimento di Chimica, Università degli Studi di Pavia, Viale Taramelli 12, 27100 Pavia, Italy; § BIOGEM Istituto di Ricerche Genetiche “G. Salvatore”, via Camporeale, 83031 Ariano Irpino, Italy

## Abstract

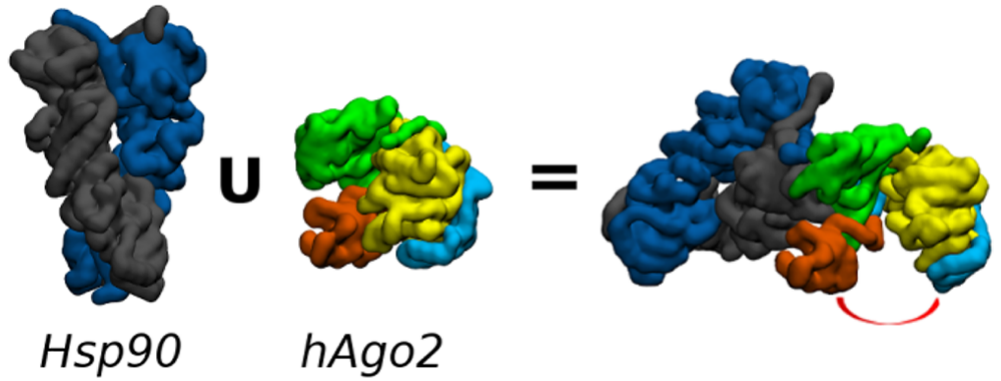

The assembly of RNA-induced silencing complex (RISC) is a key process in small RNA-mediated gene silencing. Loading of small RNAs into Argonaute (Ago), the key player protein in the process, has been shown to depend on the Hsp90 chaperone machinery. Experimental single-molecule data indicate that ATP binding to the chaperone facilitates the conformational changes leading to the open state of Ago essential to form a complex with small-RNA duplexes. Yet, no atomic-level description of the dynamic mechanisms and protein–protein interactions underpinning Hsp90-mediated Ago conformational activation is available. Here we investigate the functionally oriented structural and dynamic features of Hsp90-human Ago (hAgo2) complexes in different ligand states by integrating protein–protein docking techniques, all-atom MD simulations, and novel methods of analysis of protein internal dynamics and energetics. On this basis, we develop a structural-dynamic model of the mechanisms underlying the chaperone-assisted human RISC assembly. Our approach unveils the large conformational variability displayed by hAgo2 in the unbound vs the Hsp90-bound states. In this context, several hAgo2 states are found to coexist in isolation, while Hsp90 selects and stabilizes the active form. Hsp90 binding modulates the conformational plasticity of hAgo2 (favoring its opening) by modifying the patterns of hAgo2 intramolecular interactions. Finally, we identify a series of experimentally verifiable key sites that can be mutated to modulate Hsp90-mediated hAgo2 conformational response and ability to bind RNA.

## Introduction

Small RNAs—small interfering RNAs (siRNAs) and microRNAs (miRNAs)—can silence the expression of their complementary target messenger RNAs (mRNA) through the formation of the effector RNA-induced silencing complex (RISC). The effector complex, in turn, fine-tunes gene-regulation in diverse organisms contributing to cellular homeostasis in a number of diverse physiological processes. The Argonaute (Ago) family of proteins is the core component of RISC, acting as the catalytic engine for the endonucleolytic cleavage of target mRNAs (known as RNA interference [RNAi]) and/or as a molecular platform for their translational repression, deadenylation, and degradation.^1−3^

RISC assembly can be divided at least into two main successive steps: (1) duplex loading, in which small RNA duplexes are inserted into Ago proteins to form pre-RISC, and (2) passenger ejection or unwinding, in which the two strands are separated within the Ago protein and one of them is ejected from Ago. The resulting functional complex is called mature RISC or simply RISC.

The binding between mRNAs and Ago takes place through several contact sites. Alanine scanning experiments and spectroscopic analyses allowed to identify the N-domain of human Ago2 (hAgo2) as the initiator of duplex unwinding during RISC assembly. This event was shown to be coupled to N-domain conformational changes:^4^ indeed, the human N-domain can assume different poses in the context of the full length protein depending on the exact step of RISC assembly, similarly to what already observed in bacterial structures for the relative positioning of the Ago N-domain in the different states of the complex (apo, binary bound to the RNA guide or ternary in a protein-guide-target complex).^5−7^ Deletion of amino acids 53–135 compromises the stability of the protein, which is still able to load siRNA albeit more slowly than WT, while no detectable mature RISC (unable to unwind) is observed.^8^

Loading of small RNAs into Argonaute has been shown to require the intervention of the chaperone machinery, which entails heat shock proteins Hsp70 and Hsp90: the relevance of chaperones during duplex loading has been confirmed in different species and isoforms (fly Ago1 and Ago2, mammalian Ago2, and plant Ago1, Ago4 and Ago7).^9−15^

The current model of functioning is based on the hypothesis that Hsp90 stabilizes a high-energy, RNA-free Argonaute, which upon loading of a duplex, unwinding, and ejection of the passenger strand, stabilizes in a low-energy, guide-RNA-sequestered conformation, whereby the latter steps are likely favored by the intrinsic energy difference between the high-energy RNA-free Argonaute and a mature, guide-bound RISC.^3^

Recent studies have demonstrated that the overall domain organization of Ago proteins is highly flexible in their RNA-free form. In particular, it is hypothesized that in the absence of the chaperone machinery, the binding cavity of Ago2 is too narrow to accommodate small RNA duplexes, as observed in studies of the fly protein.^16^ ATP hydrolysis by the heat shock protein triggers the set of conformational changes needed by Ago proteins to load otherwise too bulky and sterically hindered RNA duplexes. Yet, chaperones are dispensable in the subsequent steps of RISC cycle, i.e., binding, cleavage, and release of the complementary target RNAs by RISC.^16−18^ In this framework, besides inducing a set of structural changes in the client protein Ago, the chaperone machinery itself undergoes a set of structural changes tightly connected to specific steps of its ATP hydrolysis cycle.^19^ The strong crosstalk between the mode of action of the chaperone system, its own structural behavior, and the structure/thermostability of client proteins is currently a hot subject of research and debate.^20,21^

In mammalian RISC assembly, duplex loading is an ATP-dependent reaction and Hsp90β binds to the client in its ATP-bound form.^22,23^ Recent evidence showed that the ATPase mutant E42A, unable to hydrolyze ATP, significantly reduced target cleavage activity highlighting an additional point of chaperone control over RISC dynamical assembly.^24^ Importantly, the *in vitro* reconstitution of the chaperone-mediated Ago2-RISC assembly has been extensively described for *Drosophila melanogaster* by Tomari and co-workers.^12,18,25^ Single-molecule analysis provided information on the conformational ensembles of the intermediate states in RISC biogenesis and target cleavage mechanisms. Moreover, the authors gained insights into the mammalian assembly of human RISC further confirming the high conservation of the duplex loading mechanism and the requirement for the intervention of chaperones to incorporate bulky RNAs into Ago.^24^

Despite these fundamental advances, understanding the structural basis of chaperone-mediated RISC assembly, in other words how Hsp90 selects, binds and stabilizes the conformation competent for duplex loading (the active state), represents a challenging task. The structural organization of this state remains elusive, and no atomistic-resolution data are available concerning domains or regions of Argonaute that are connected by chaperones. Moreover, questions still remain on which of these interactions are essential for determining the active form of Argonaute and for RNA-loading.^3,18^

The aim of this work is to develop a structural model that can provide atomistic insights into the mechanisms underlying the chaperone-assisted human RISC assembly, rationalizing experimental evidence, and laying the foundations for the design of approaches to control/tune RNA silencing processes. To achieve these goals, here we combine protein–protein docking calculations with extensive MD simulations and novel methods of analysis of structural dynamics and energetic stabilization of complexes to generate a model of the Hsp90 and human Ago2 complex, recapitulating the determinants of the functionally oriented aspects of the interaction. Moreover, we present a detailed investigation of the internal dynamics of hAgo2 free and hAgo2 in complex with the chaperone. In this framework, we pinpoint hAgo2 single-point mutants acting as key hotspots driving the interaction with Hsp90 that may ultimately modulate RNA loading mechanism. Our computational approach offers new insights into the role of Hsp90 in RISC assembly and contributes experimentally verifiable key sites for the investigation of Argonaute-mediated gene silencing mechanisms.

## Results and Discussion

### Structures Examined in This Study

**Figure 1:**
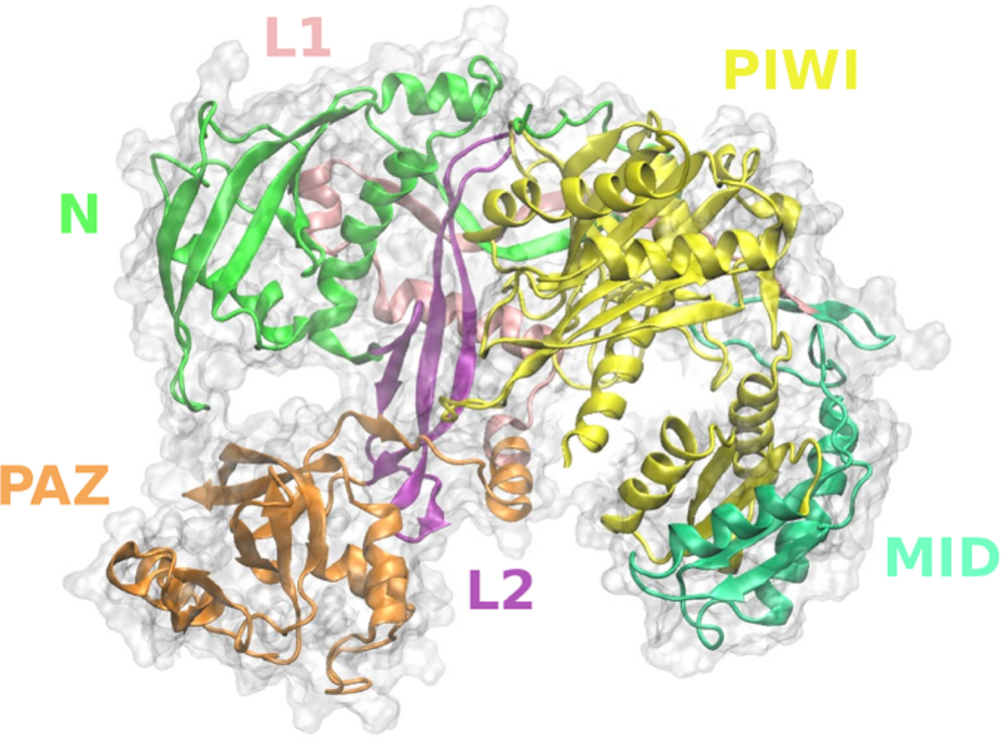
Human Argonaute. 3D structure of hAgo2 is shown in cartoons and ghost surface. N-terminal, PAZ, MID and PIWI, connected by two linker domains (L1 and L2), are indicated.

Recently, the crystal structure of human Ago2 bound to a mixed population of siRNA was solved, showing that the overall domain organization and mode of siRNAs binding are similar to those of prokaryotic Argonautes, which have been more extensively studied (Figures 1 and S1).^1,6,7,17,26−30^ Herein, small RNA binding plays an important role, locking the flexible apo form of the eukaryotic Argonaute protein into a single dominant conformation. Indeed, the high conformational variability of apo hAgo2 is likely the reason why no X-ray structure of the full length apo protein has been determined to date.^17,30,31^

From a structural point of view, hAgo2 consists of four conserved domains: N-terminal, Piwi/Ago/Zwille (PAZ), MID, and P-induced winpy testis (PIWI), connected by two linker domains (L1 and L2) (Figure 1). Overall, the protein appears arranged in two main lobes, where the nucleic acid-binding channel runs across the four domains. The loaded strand serves as the spine of the Ago protein: limited proteolysis with thermolysin suggested that, in the absence of the RNA strand, the MID domain is hinged toward the PIWI domain and adopts an open conformation whereby the interface between the two is exposed to solvent while the C-terminal region remains unfolded.^15,17,28^

On the other side, Hsp90 displays a multipartite structure where different domains control distinct functions, connecting ATP processing in the N-terminal domain and client binding/remodelling in the middle domain. Recent results unveiled an intricate array of correlated functional motions among distant regions of Hsp90 depending on specific binding states, which helped gain insights into the complex mechanisms used by the chaperone to assist the formation of multiprotein assemblies.^21,32−35^ In this scenario, investigating the internal dynamics pattern of the chaperone-hAgo2 assembly can shed light on the traits of internal dynamics of the complex that are connected to its functional role in the RISC conformational cycle.

### Building Models of the hAgo2-Hsp90 Complex

We first set out to characterize the conformational ensemble of the apo protein (apo hAgo2) to detect distinct dynamic and energetic properties of the unbound state that may be perturbed upon Hsp90 binding. Extensive all-atoms molecular dynamics (MD) of the unbound hAgo2 protein were run at different temperatures, 300, 350, and 400 K. Nonphysiological temperatures were used to speed up structural sampling toward local unfolding events and expand conformational ensembles. Importantly, it has been shown that the highly dynamic character of the apo form of hAgo2 is dramatically reduced when small RNAs bind the protein and that this process is assisted by the chaperone machinery.^16^ In order to investigate the effects of the chaperone on Ago internal dynamics and their effect on Ago structural modulation, we modeled the structural complex of the interaction between Hsp90 and hAgo2 and studied the dynamic and energetic patterns of the complex. As no direct experimental knowledge on the interacting faces of the two proteins is available to guide the docking simulation of hAgo2-Hsp90 complex, we run different (blind) predictions to select the representative complex.

It is worth noting here that the Hsp90 structure selected for these calculations is the closed one, which corresponds to the chaperone conformation in complex with the Cdc37 cochaperone and the Cdk4 client kinase in the cryoEM structure solved by the Agard group. As an important caveat, it must be mentioned that alternative, more open, Hsp90 conformations could have been used for our calculations. In this picture, the chaperone would offer larger contact surfaces to the recognition of hAgo2. Our working hypothesis here is that this Hsp90 conformation corresponds to the most likely activated state, responsible for productive binding and reshaping of clients, as observed in the Agard structure. In this framework, we reasoned that running our docking experiments on open Hsp90 structures modeled on SAXS or FRET data may have suffered from limitations due to low resolution and introduced a level of variability that could expectedly have affected the results.

**Figure 2:**
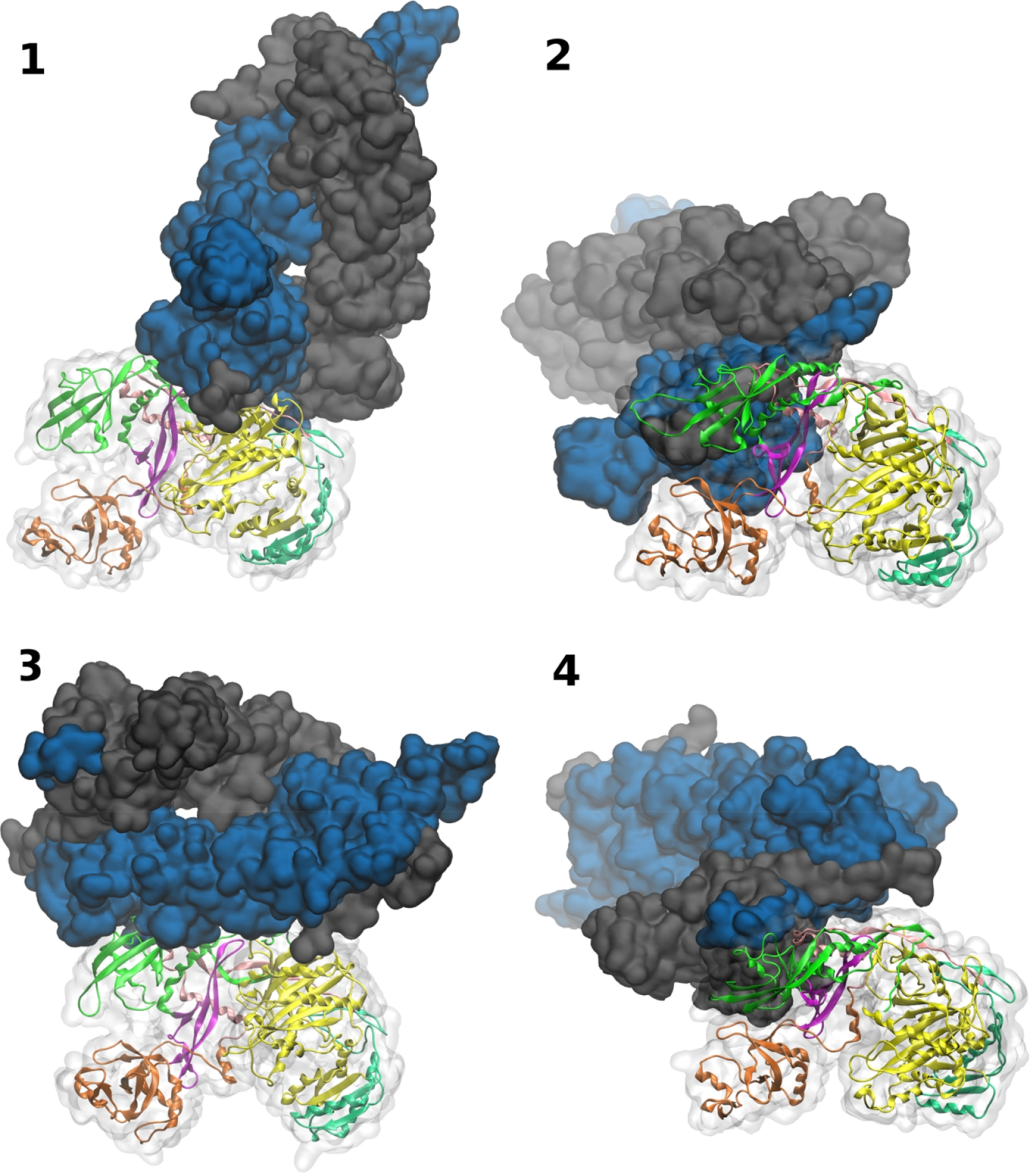
hAgo2-Hsp90 complexes. Selected docking poses for hAgo2-Hsp90 interaction (see Methods). hAgo2 is shown in cartoons (N → green, PAZ → orange, PIWI → yellow, MID → lime, L1 → purple, L2 → pink) and ghost surface. Blue and gray solid surface representation is used for Hsp90 subunits.

Specifically, predictions of hAgo2-Hsp90 complex were carried out using a combination of docking methods, ClusPro, Zdock, and Patchdock, based on rigid-body protein–protein protocols (see Methods): the results of the three different approaches were combined to identify consensus complex poses by in-depth structural analyses, visual inspection, and evaluation of consistency with available data. At the end of the process, four different models of the hAgo2-Hsp90 complex were selected (see Methods), where Hsp90 preferably binds the N-domain of hAgo2, consistent with recent experimental evidence on hAgo2 N-domain mutants with compromised RISC cycle, but no direct association with chaperone-mediated conformational opening.^8^ In three out of four models (complexes **1**, **2**, **4**), at least one of the Hsp90 N-domains is engaged in a stable interaction with hAgo2 (top interactions, Figures 2 and S2). In complex **1** Hsp90 N-domains interact with residues of PIWI domain, whereas in complexes **2** and **4** the binding concerns hAgo2 N-domain (Figures 2 and S2). In the remaining model (complex **3**), one of the two Hsp90 M-domains binds the hAgo2 N-domain, resulting in a highly asymmetric unit (side interaction, Figures 2 and S2).

### Characterization of the Structural Dynamics of the Different Models and Comparison with the Apo state: Functional Implications

**Figure 3:**
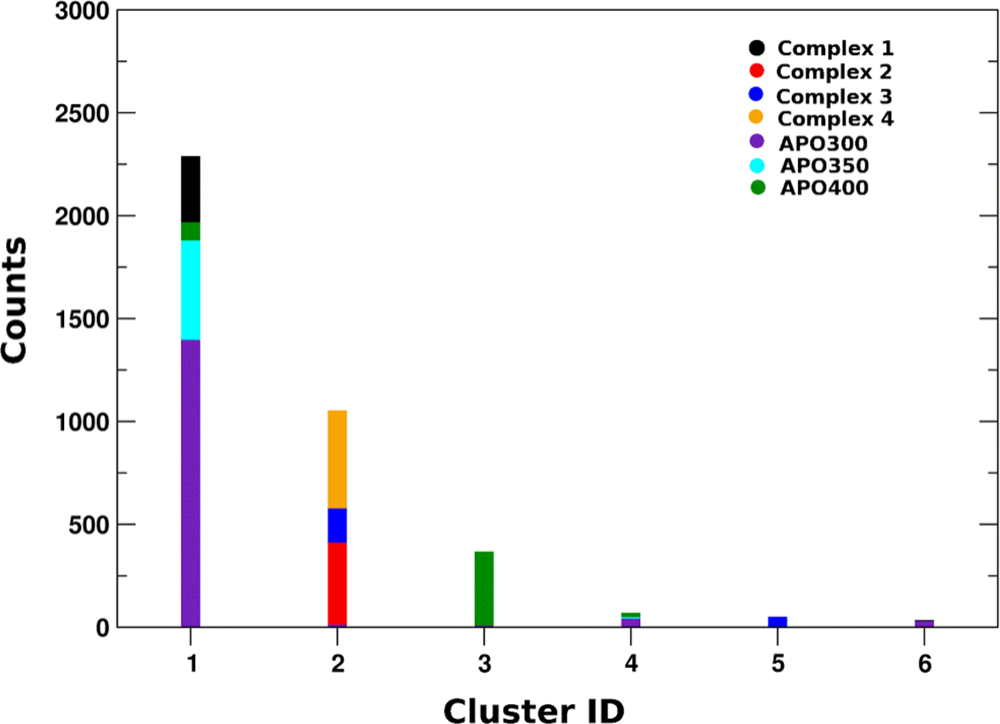
Cluster analysis on hAgo2 meta-trajectory. Cluster id and counts per cluster are indicated on *x*- and *y*-axes, respectively. Cluster composition is displayed and colored per system. See Methods.

Extensive MD simulations were run for each predicted complex to gain insights into the structural stability of the complexes and/or evaluate the occurrence of local rearrangements. The dynamic evolution of the unbound hAgo2 at different temperatures and in complex with Hsp90 immediately shows the extremely flexible nature of Ago, which is modulated by chaperone binding. To clarify the dynamic behavior of hAgo2, we built a unique hAgo2 meta-trajectory obtained by the concatenation of all the single MD runs from both the apo and the Hsp90-bound states. Cluster analysis (performed applying the Gromos clustering algorithm^36^) was applied to the meta-trajectory, considering the backbone atoms of hAgo2 (RMSD cutoff set to 0.5 nm, to account for the expectedly large structural variations of the protein). Figure 3 shows that in the absence of Hsp90, hAgo2 largely adopts one preferential conformation (cluster #1), which corresponds to a closed structure not competent for RNA loading. In contrast, in hAgo2-Hsp90 complexes, hAgo2 substantially populates distinct conformational ensembles, with the exception of complex **1**, for which the conformations collected in cluster #1 (corresponding mostly to the apo state) are accessible. Indeed, complexes where hAgo2-Hsp90 interaction takes place at the hAgo2 N-domain (complexes **2**, **3**, and **4**) share common structural dynamics traits, with Ago populating mainly cluster #2, representative of open conformations of the client. Moreover, a secondary effect related to the orientation (top/side interaction) of hAgo in the binding pose can be observed. While complexes **2** and **4** stably adopt cluster #2 conformations, complex **3** shows larger dynamic variability. These preliminary investigations of the hAgo2-Hsp90 interaction models suggest that Hsp90 can stabilize a set of open hAgo2 conformations that may be only marginally visited by the apo protein.

Specifically, it is worth noting that among the four hAgo2-Hsp90 complexes, complex **1** appears to be the least compatible with experimental/biochemical observations: in this model, Hsp90 does not significantly affect hAgo2 dynamics compared to the apo state, stabilizing a conformation that is largely superimposable to the unbound state. Complex **3** represents an intermediate case; even though chaperone binding modulates the dynamic behavior of hAgo2, compared to the apo form, the observed high conformational plasticity of the complex (given the occupancy of clusters **2**, **4**, **5**, and **6** in Figure 3) indicates a relatively unstable interaction. In this framework, complexes **2** and **4** appear to be the models that best capture, at a qualitative level, the general features of Hsp90 and hAgo2 binding that may be related to the functional properties of the complex. In particular, the two models suggest a role for Hsp90 in the preferential selection of stable hAgo2 open structural states, preorganized for efficient polynucleotide binding. This result is consistent with the experimental observations that point to the presence of the chaperone as a necessary requirement for RNA loading by the client. It is tempting to suggest that Hsp90s role is to stabilize a functional conformation of the client that would not be populated in the uncomplexed state. The chaperone does not act as a foldase but rather as a regulator/promoter of the functions of other proteins.^8,16^

**Figure 4:**
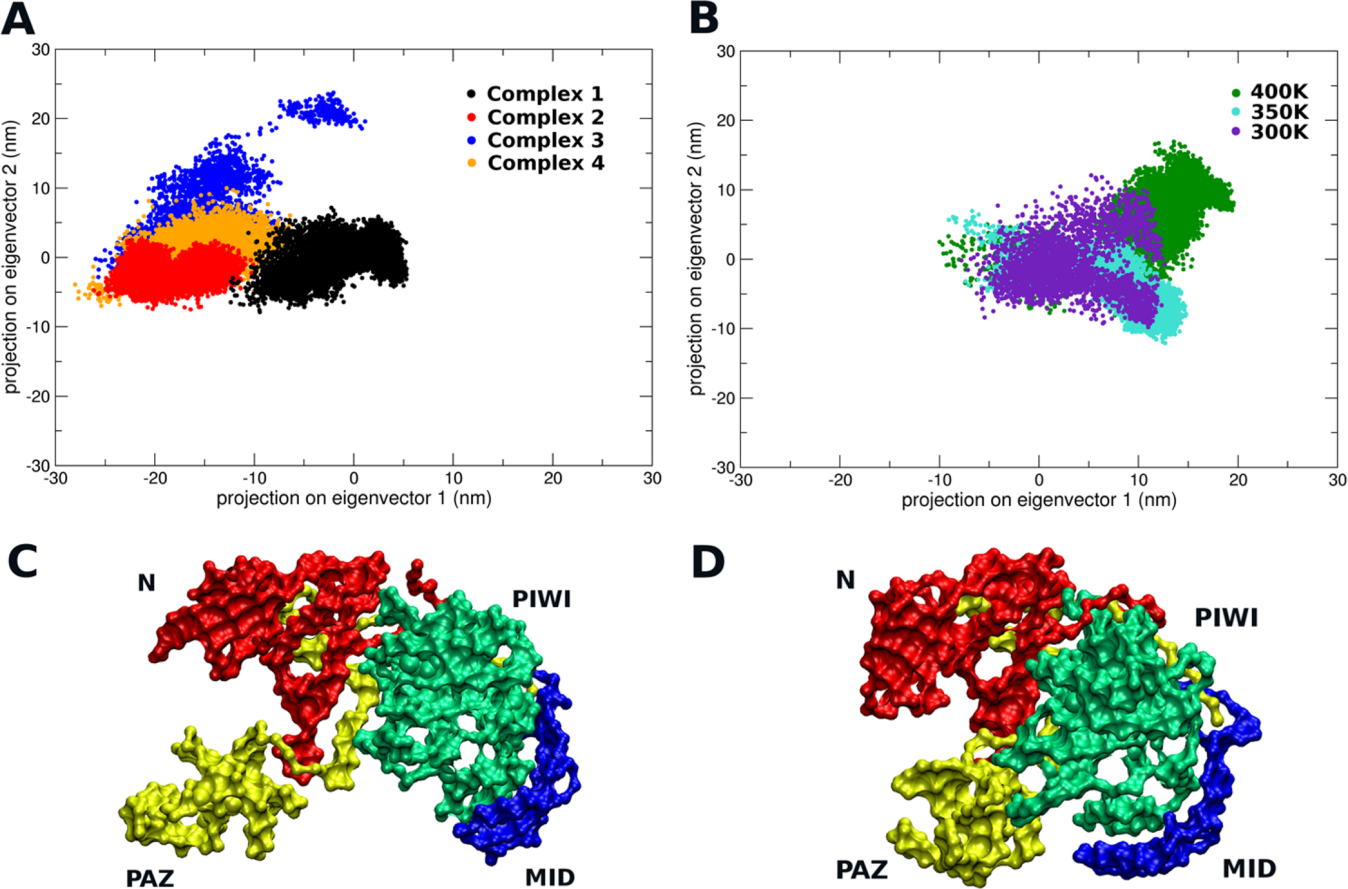
PCA analysis. Principal component analysis on hAgo2 in the complexes (A) and at different *T* (B). hAgo2 covariance matrix has been built on Cα atoms and used for individual system trajectory projection. The first two principal components (eigenvectors 1 and 2 on *x*- and *y*-axes) are used for the projection. Extreme projections along the meta-trajectory on the average structure corresponding to hAgo2 in the complexes (C) and apo hAgo2 (D) are rendered in surface views.

To characterize the main protein motions explored in the different cases, we next performed a principal component analysis on hAgo2 protein (Cα atoms) along the meta-trajectory (Figure 4). First, PCA confirms that, once promoted by the contact with Hsp90, the open states of hAgo2 are stable: indeed, in complexes **2** and **4**, hAgo2 spans a limited ensemble of conformational space. In contrast, apo hAgo2 is characterized by a much broader distribution of conformations (colored spots in Figure 4). We observe that the presence of Hsp90 pushes hAgo2 to sample different regions of the essential space, compared to the unbound case (different values of the first vector describing the principal motions on *x*-axis). Along these lines, the temperature increase in apo hAgo2 simulations favors the transition of the protein to compact structures where the RNA cleft is narrowed and PAZ and MID domains can freely move closer to each other. In contrast, even at higher temperatures, hAgo2 explores more open conformations, competent for duplex loading, in association with the chaperone.

**Figure 5:**
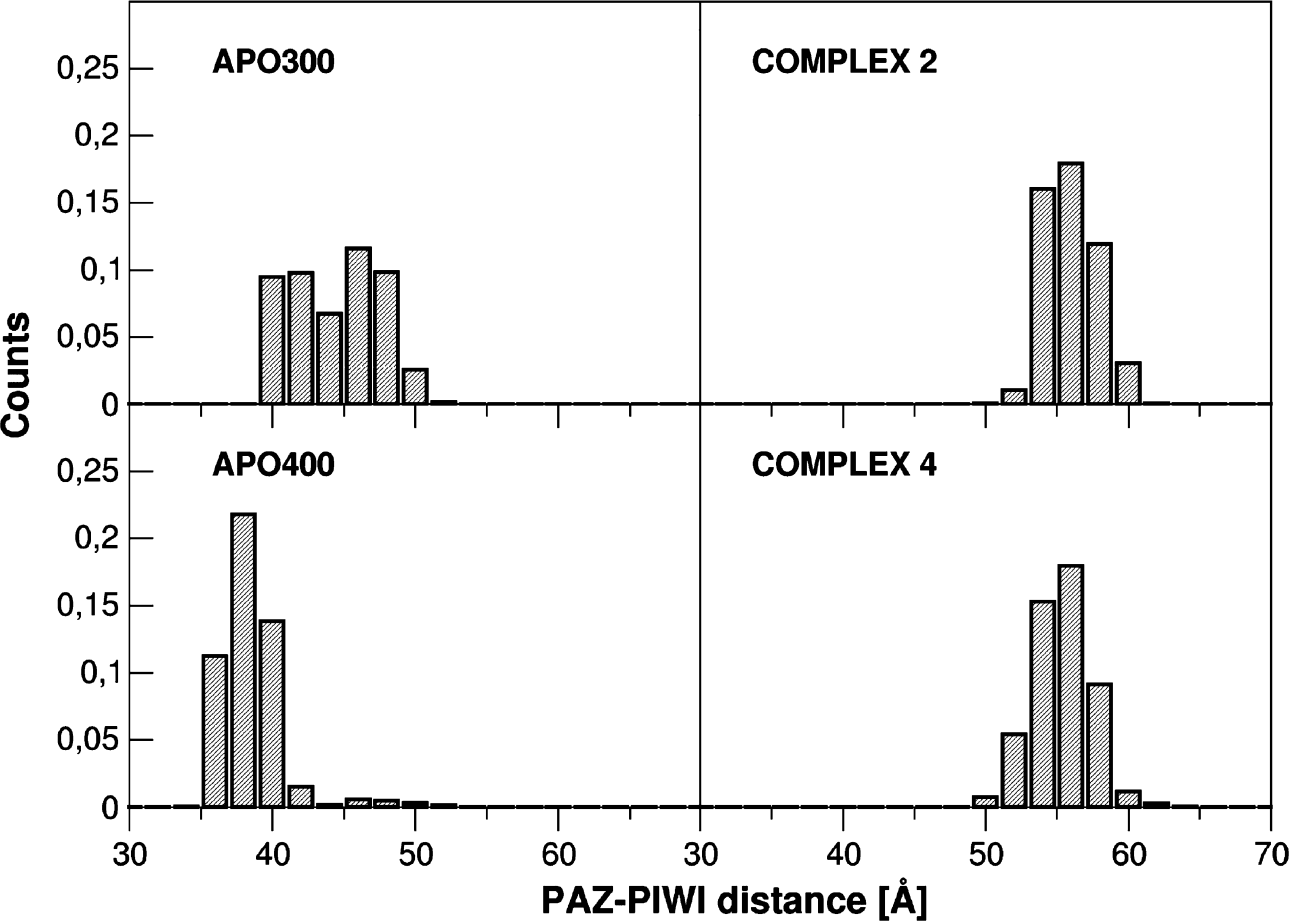
PAZ–PIWI distances. Statistical distribution of the distances between the center of mass (COM) calculated on PIWI and PAZ domains (see Methods) along the simulation time of hAgo2 in the apo form at 300 and 400 K and bound to Hsp90 in complex **2** and **4**. On the *y*-axis, histograms counts are normalized to 1.

At the domain level, the differential dynamic modulation induced by Hsp90 is reflected by the diverse structural domains rearrangements of hAgo2. Specifically, we measured the distance between the center of mass (COM) of PAZ and PIWI domains along the simulation time, as an index of the reciprocal positioning of the two lobes. This analysis (further confirmed by the evolution of the angle spanned by the two lobes of Ago protein, see Figure S3 in the Supporting Information) demonstrates that the presence of the chaperone favors the opening and “freezes” Ago in a structural organization where the two lobes are well separated (Figure 5). This wider conformation presents a larger cavity for RNA recognition and loading. In this context, a broader distribution of COM distances is further observed for the apo hAgo2 simulations (300 K), as shown in Figure 5, consistent with previous analyses. Importantly, the larger COM distances we report are qualitatively consistent with experimental distances (56 Å) measured by FRET analysis between PAZ and MID-labeled domains in the Ago2-RISC, where small RNA loading prompts Ago2 to adopt a more open conformation.^17,18^

### The Determinants of Differential Dynamics: Insights from Energetic Analysis of hAgo2 in Different Complexes

As the presence of Hsp90 is shown to tune the global conformational dynamics of hAgo2, we investigated whether distinct internal patterns of residue-pair interactions can be identified for hAgo2 in the complexes and in the apo form. The underlying hypothesis is that differential combinations of pair interactions may stabilize hAgo conformations in isolation or in the complex. The functional relevance of distinct specific patterns of predicted intraprotein interactions determined by Hsp90 being bound or unbound is experimentally verifiable by, e.g., site-directed mutagenesis and analysis of mutation impact on the stability of the complexes.

To this end, we have used the energy decomposition method (EDM).^37,38^ Our approach is based on the concept of domains as compact and independent folding units (i.e., stability cores) and on the analysis of the residue–residue energy interactions obtainable through classical all-atom calculations. In particular, starting from the analysis of the residue-based nonbonded pair-interaction energy matrix associated with a protein, our method filters out and selects only those specific subsets of interactions that define possible independent stability cores within a complex protein structure. This allows grouping different protein fragments into energy clusters that are found to correspond to the stabilization cores of domains in specific conformations. EDM is in fact designed to identify specific regions that contribute the most to the stabilization of a certain conformational ensemble, through eigenvector decomposition and simplification of the residue pair-interactions matrix.

**Figure 6:**
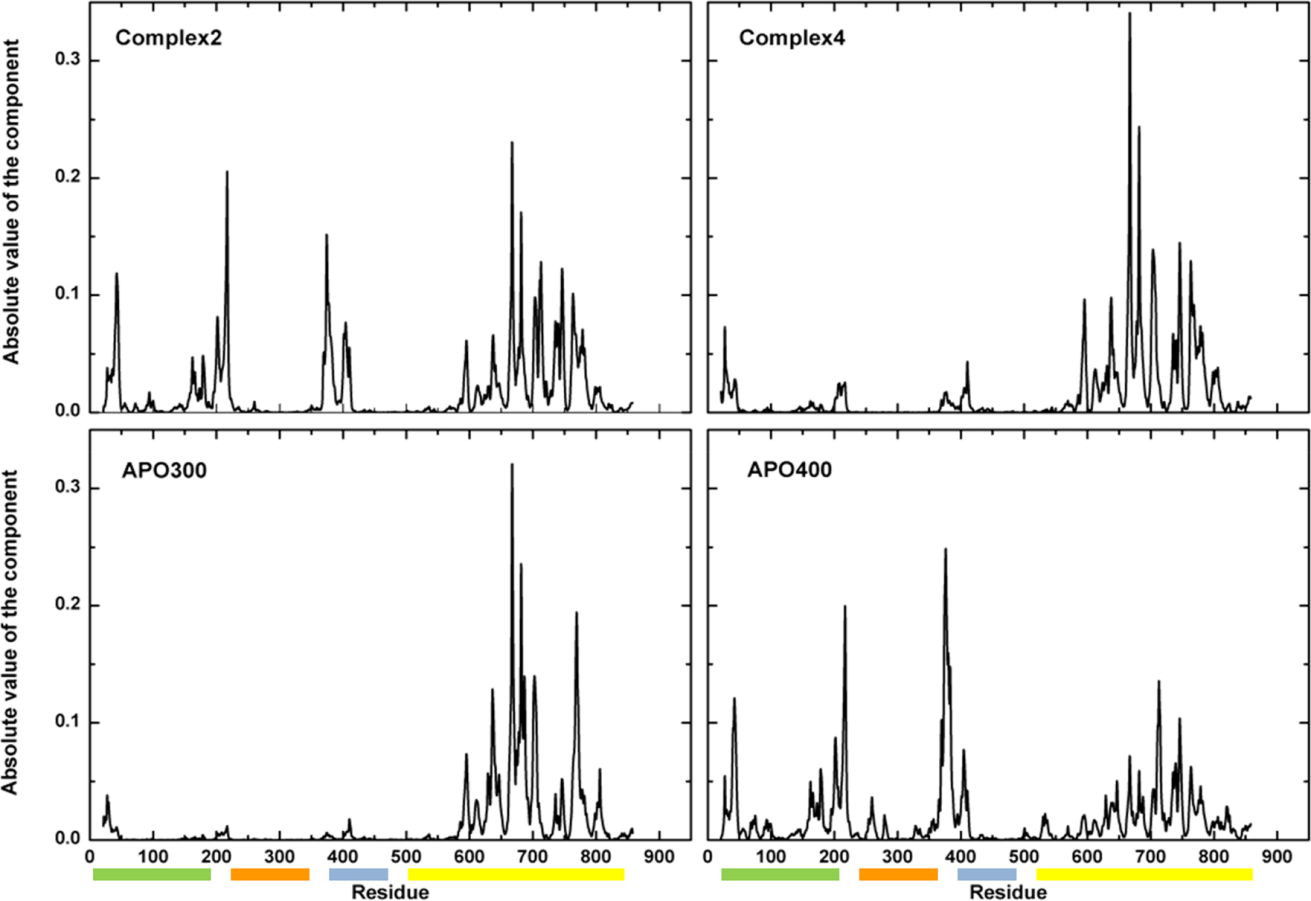
EDM profile. hAgo2 nonbonded interactions energy along the sequence described by the first component. Higher peaks correspond to the regions that recover most of the stabilization energy. On the *x*-axis, hAgo2 domains are indicated as colored bars along the following sequence: N-domain (green), PAZ (orange), MID (gray), and PIWI (yellow).

Comparative analysis of EDM matrices for the apo and bound forms demonstrate that Hsp90 binding reorganizes the energetics of internal interactions in hAgo2 (Figure S4). Upon complex formation, we observe a redistribution of the stabilization nuclei that eventually span various regions of hAgo. New hotspots (brighter points) of increasing stabilization appear on top of the PIWI domain. To identify which residues contribute the most to the new energy pattern in the complex, we analyzed the components of the eigenvector that recapitulates most of the nonbonded energy (this eigenvector is the one associated with the first eigenvalue of the EDM matrix; see Methods). In the Hsp90-bound conformations, the stabilization energy distribution for hAgo redistributes from PIWI (that represents the principal stability core in the apo state at room temperature) to other regions. In particular, in complex **2** (and to a smaller extent in **4**) Hsp90 appears to favor the formation of stability cores in the N, PAZ, and MID domains, at the expense of the PIWI region (Figure 6). In this view, a more diffuse organization of stabilizing interactions, induced by the chaperone, may facilitate access to open hAgo2 states alternative to the closed ones observed in the apo state. More open conformations ought to be expectedly more prone to RNA binding.

Biochemical studies demonstrate that small-RNA loaded hAgo2 is more stable than empty Ago.^17^ From our analyses, the perturbation triggered by RNA binding that involves mainly PIWI and PAZ domains could significantly impact on the whole protein stability in a state where the stabilizing nucleus is mainly located in the PIWI domain. In contrast, chaperone binding may activate an energy redistribution that results in a more plastic system capable of functional adaptation in which the interaction with RNA would not compromise the global stability.

### Connecting Internal Energetics and Dynamics: Implications for Recognition Mechanisms

On the basis of these findings, we next asked whether this energetic modulation could be associated with specific internal dynamic properties. In order to gain insight into the intrinsic dynamics and flexibility of the protein, we analyzed the residue-pair distance fluctuation along the simulation time (see Methods), which allows the identification of patterns of coordination between physically distant regions.

The presence of Hsp90 also reverberates on the internal dynamics of hAgo2. The yellow areas in Figure S5, associated with flexible regions, decrease when the chaperone binds the client. In particular, this event significantly reduces the flexibility of PAZ domain. This differential modulation becomes clearly evident when a principal component analysis is performed on hAgo2 Cα atoms in each system. The characterization returns a remarkable difference of PAZ dynamics for the unbound and complexed Ago systems (see Figure S6). The presence of the chaperone, anchoring PIWI and N domains, limits their dynamics with respect to the unbound form, especially for the N domain. This may favor a coordinated motion of N and PAZ domains, as indicated in the matrix obtained as the difference between the DF patterns in the unbound and complex states (see Figure S5), where regions identifying protein residues that increase the dynamical coordination upon Hsp90 binding are evidenced. It is interesting that also the reciprocal motion of PAZ and MID domains turns to be affected by the presence of the chaperone. The internal dynamics analyses suggest indeed that the stabilization of a more open accessible Ago state is driven by the reduced flexibility of PAZ domain, that is linked to a decreased flexibility of the N and more importantly MID domain, where the first event of RNAs anchoring is known to occur. This result directly links chaperone mechanisms to hAgo2 RNA binding and loading.

**Figure 7:**
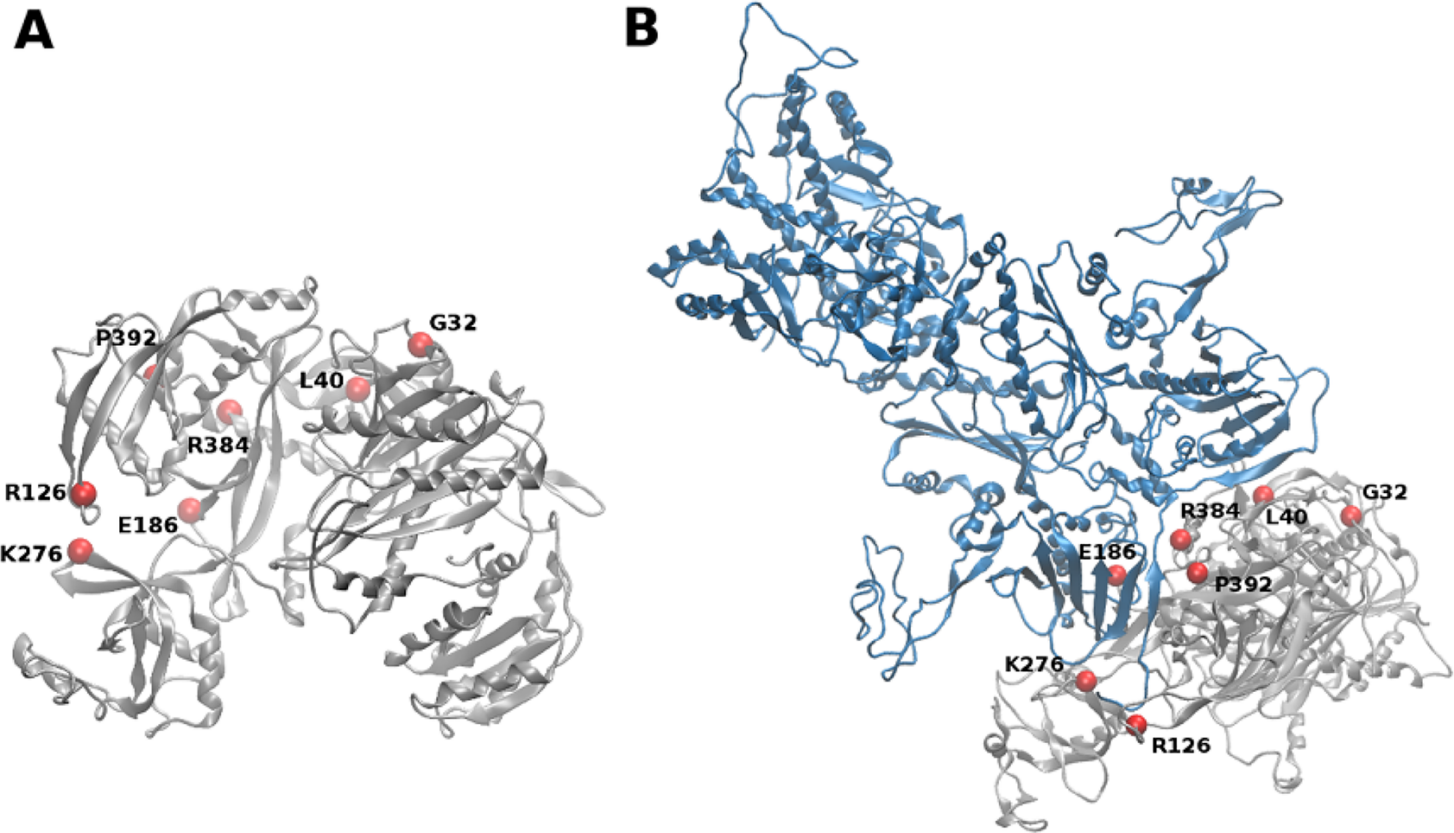
hAgo2-Hsp90 complex. Localization of the computed hotspots (red spheres) mapped onto hAgo2 in the unbound state (A) and in the complex with Hsp90 (B). hAgo2 and Hsp90 are displayed in gray and blue cartoons, respectively. For clarity, only complex **2** is displayed.

Taken together, the differential energetic and dynamics fingerprints of the protein in the various states were used to guide the definition of those areas of the interacting surfaces that may be crucial in the chaperone recognition process. From the analysis of interacting regions of hAgo2 and Hsp90 in complex **2** and **4**, we defined a consensus stretch of amino acids present in both interfaces: in particular, hAgo2 residues bonded to Hsp90 were selected and analyzed. Hence, by combining these hotspots on the common interface between complexes **2** and **4** with oncogenic mutational mapping from the COSMIC database (https://cancer.sanger.ac.uk/cosmic), we were able to identify specific residues that have a high probability of modulating hAgo2-Hsp90 interaction, whose mutation results in a phenotypic functional impact. Specifically, we focused on those amino acids at the interface that are involved in key contacts (salt bridges or hydrogen bonds interactions) between the two binding partners and that played a special role in complex stabilization (see also Figure 6). We identified as important interactors residues R126 and E186 from the hAgo2 N-domain, and residues K276, R384, and P392 in the PAZ domain (Figure 7), which are found to correspond to oncogenic point mutations in the COSMIC database.

To further validate our predictions, we investigated additional N-domain hAgo2 mutants that have been proven deficient in RNA-duplex loading (chaperone-dependent activation) from *in vitro* RISC assembly experiments.^7,8^ Thus, we modeled G32A, L40A, R126A, E186A, K276E, R384E, and P392K mutants and analyzed the impact of these mutations on protein complex stability (ΔΔ*G*). Two structure-based predictors were used, I mutant (http://folding.biofold.org/i-mutant/i-mutant2.0.html) and SDM (http://marid.bioc.cam.ac.uk/sdm2/), both taking advantage of the large structural information available on the effect of single point mutations on native protein folds (ProTherm and Homstrad+Toccata databases, respectively). I mutant is a neural-network-based approach able to estimate changes in protein stability from either sequence or structure descriptors, relying on an energy-based FOLD-X algorithm.^39^ The second method, SDM, is based on a statistical potential energy function derived by environment-specific substitution frequencies tables, providing a stability score prediction and estimation of disease association.^40^

This cross-validation revealed that the two major and opposite effects concern L40A, as the most destabilizing substitution for both predictors (ΔΔ*G* = −2.3 kcal mol^–1^ (I mutant) and −3.9 kcal mol^–1^ (SDM) on average for the two complexes) and K276E as the least destabilizing mutant. It is worth underlining that K276E is predicted to slightly stabilize the complex (SDM) or very unlike to occur (as indicated by a specific reliability score provided by I mutant).^39,40^ The remaining mutations exhibit an intermediate behavior between these two cases, yet displaying some destabilization effect. Protein stability changes upon mutations are given in Table S1: normalized ΔΔ*G* values calculated by the two servers are given to improve comparability and point out relations between the two (Figure S7). SDM and I mutant showed a good correlation in the predictions of energy changes, with *R*^2^ = 0.82 and *R*^2^ = 0.9 for complexes **2** and **4**, respectively: indeed, mutations on complexes **2** and **4** yielded to very comparable trends, whereby E186A and R384E, localized at the hAgo2 L1 and L2 linkers respectively, turned out as the more difficult point mutations to predict, likely because of their interfacial position.

In light of these findings, all the designed mutations seem to alter the complex stability except K276E. This result can be explained by the high solvent accessibility in this position: the smaller impact on the complex could rely on the enhanced conformational freedom of the side-chain.

Summarizing, the detailed analyses of the complex interfaces have highlighted critical points of interaction between the two binding partners. Consistently, one of the most destabilizing mutations predicted, G32A mutant, showed a large defect in loading the siRNA duplex, lower for microRNA duplex, supporting recent observation that the more flexible RNA duplex (mismatch-containing microRNA) is slightly less dependent on the Hsp90 ATPase activities compared to the perfectly complementary and more rigid siRNA duplex, which would require a complete/functional hAgo2 opening. This picture provides a structural basis to the effect of the G32A mutant, which can be reconnected to a defect in the chaperone-dependent activation.^8,24^

At this stage and based on the limited knowledge concerning the hAgo2-Hsp90 complex, though we cannot rule out the possibility of a different structural rearrangement between the two proteins, these data seem to corroborate the reliability of the designed model interface: additional support to our comparative approach may be seen in the chaperone mutational profile reported by the COSMIC database, where Hsp90 amino acids involved in hAgo2 key interactions show a high degree of mutability (i.e., Y56C, N101Y, T110A, H149Y, D151N, H171Y, D228N, and E232K).

## Conclusions

By combining protein–protein docking techniques, extensive MD simulations, and novel methods of analysis of protein conformational stability, we have developed a structural model of the chaperone-assisted RISC assembly that proves able to recapitulate the salient traits of dynamics and interactions underlying complex formation. It is important to stress that the simulations and analyses of internal energetics discussed here have been based on the use of one specific version of the Amber force field.^41^ Recent studies have underlined the dependence of dynamic features in NMR ensembles of protein conformations. While it would be desirable to test the models of the large complexes described here with different force fields, it is important to underline that our calculations show a clearly differentiated dynamics of hAgo2 in the absence or presence of Hsp90. In this context, it is important to underline that the models are able to unveil hotspots whose functional relevance had already been proven experimentally. Being corroborated by independent experimental findings, our results provide an atomic-resolution mechanistic view on the roles of Hsp90-hAgo2 binding in RNA loading. This model demonstrates that the presence of the chaperone is able to modulate the conformational plasticity of hAgo2 favoring its opening. Such motion is compatible with a state of the protein primed to load its substrate, thus capable to bind RNAs. Moreover, our model demonstrates that Hsp90 binding modifies the energetic patterns of hAgo2: we could identify the regions of the interacting surfaces essential for a stable interaction. In this framework, we defined single-point mutations that could potentially compromise/weaken the recognition mechanism. These results pinpoint a key role for Hsp90 in RNA loading.

Recent advances in Ago2 conformational activation in *Drosophila* showed a coordinated mechanism triggered the Hsp70/Hsp90 chaperone machinery. By smFRET experiments, Tsuboyama et al.,^18^ underscored the pivotal role of Hsp70 in priming the opening of fly Ago2 with the subsequent recruitment of Hsp90, which is required to intercept and stabilize the open and effector form of Ago2 to assemble functional RISC. An equivalent function has been recently clarified for Hsp90 in the cryoEM structure of the Hsp90-Cdc37-Cdk4 complex, where the chaperone binds and stabilizes the kinase domain in a more open shape.^42,43^ Furthermore, in a previous work, we carried out an in-depth analysis of internal energy fingerprint of several kinase domain that concurred to reconcile the Hps90 ability to bind specific kinase proteins with client thermostability.^21,44,45^

From a structural perspective, our selected hAgo2-Hsp90 models (complexes **2** and **4**) display interacting surfaces that significantly overlap with the binding mode that Hsp90 shows with another client.

The comparison of our predicted models with the Hsp90-Sgt1 complex (PDB 2JKI), reveals indeed that both the clients engage Hsp90 N-terminal domain through very similar interaction sites (Figure S8B). In contrast, a different positioning is observed for the client kinase in Hsp90-Cdc37-Cdk4 complex, even if the key anchoring role of the N-domain of the chaperone in the recruitment of the cochaperone-client is maintained (Figure S8C,D). The differences in the interaction sites of Hsp90 are in line with its chaperone functionality and the broad set of different client proteins that Hsp90 binds and stabilizes. Nevertheless, our data suggest that key interactions on the N–domain could be conserved among different binding modes in different complexes.

Structures of prokaryotic Argonaute complexes show a highly flexible N-domain able to move freely with respect to the rest of the protein depending on the size of the RNA duplex bound within the central cleft.^7,8^ Furthermore, it has been proposed that this structural element might be conserved, given that a fully complementary duplex modeled onto human Ago2 structures would show a similar clash.^3^

Altogether, our data demonstrate that the stabilization of a more open hAgo2 conformation is fundamentally promoted by chaperone binding: Hsp90 could induce structural rearrangements to open hAgo2 cleft to accommodate RNA duplex or select and bind states that are only minimally present in the unbound hAgo2 conformational ensemble. Consistent with this, Jiang and collaborators identified a metastable open state of hAgo2, able to accommodate microRNA, in rapid equilibrium with other states.^46^

Our results reinforce the hypothesis that hAgo2 recruitment operated by the chaperone may occur at the very first stage of RISC cycle. In *Drosophila*, Hsp70 is dedicated to initiate the conformational opening of Ago whereas Hsp90 stabilizes the opened form; Hsp90 alone was unable to produce the active, open form of Ago. Our simulations of apo-hAgo2 carried out at different temperatures, and subsequently used to build the interacting complex with Hsp90, were designed to expand the structural ensemble of Argonaute in its unbound state and, therefore, to mimic the effect of external cofactors, such as the cochaperone Hsp70.^18^ Our interaction model reconciles the coexistence of several hAgo2 states from which the chaperone system selects and stabilizes the active state. In this light, molecular dynamics simulations highlight the large conformational variability displayed by hAgo2 in the unbound form compared to the complex, thus providing a structural hypothesis for the conformational organization of the two proteins in the complexed state. Furthermore, our studies allowed us to characterize the energetic effect driven by the interaction with Hsp90 and to suggest mutants that can be tested experimentally.

Well-tempered regulation of small RNA levels is critical for diverse biological processes in various organisms. A better understanding of the sequential dynamic conformational changes of interacting partners during RISC assembly can ultimately illuminate the molecular bases of disease mechanism and could pave the way for new strategies to tune AGO proteins toward gene silencing tools and RNAi therapeutics.

## Methods

### MD Set Up

The starting X-ray model of hAgo2 was retrieved from the Protein Data Bank with access number 4Z4C, removing target RNA. Full-length hAgo2 is made by 838 residues, namely N-domain (aa. 36–166), L1 (aa. 176–226), PAZ (aa. 231–365), L2 (aa. 374–420), MID (aa. 429–511) and PIWI (aa. 496–797). Protein refinements were carried out using Maestro (Schrödinger Release 2016-4, LLC, New York, NY).

The MD simulation package Amber v12^47^ was used to perform computer simulation by applying the Amber-ff99SB force field.^48^ The systems were solvated, in a simulation box of explicit water molecules (TIP3P model),^49^ counterions were added to neutralize the system, and periodic boundary conditions were imposed in the three dimensions. After minimizations, systems were subjected to an equilibration phase where water molecules and protein heavy atoms were position restrained, and then, unrestrained systems were simulated for a total of 8 μs, in a *NPT* ensemble; a Langevin equilibration scheme and a Berendsen thermostat were used to keep constant temperature and pressure (1 atm), respectively. Electrostatic forces were evaluated by the particle mesh Ewald method^50^ and Lennard-Jones forces by a cutoff of 8 Å. All bonds involving hydrogen atoms were constrained using the SHAKE algorithm.^51^

An ionic strength of 0.150 M NaCl was reproduced based on experimental settings.

To enhance sampling independent replicas of 500 ns (3 × 0.5 μs at 300 K, 0.5 μs at 350 K, and 0.5 μs at 400 K μs) were run for each system with different initial velocities at 300, 350, and 400 K temperatures.

The same MD settings were applied to the simulations of hAgo2-Hsp90 complexes and run on Acellera (ACEMD). Two independent replicas of 500 ns were run for each complex (4 complexes × 0.5 × 2 = 4 μs).

MD analyses were carried out on a meta-trajectory (heavy atoms), obtained by concatenating all the trajectories of the apo form of hAgo2 at different temperatures and the hAgo2 from hAgo2-Hsp90 complexes.

Figures are created using VMD, Pymol, and Chimera.^52−54^

### Hsp90-hAgo2 Complex Model

#### Protein–Protein Docking

Docking experiments were carried out using the refined Hsp90 structure from the cryoEM complex (5FWK pdb access code^43^) and different RNA-free hAgo2 structures (X-ray structure, PDB code 4Z4C,^30^ and two representative structures of the open and closed form from MD simulations) in order to consider the large structural variability displayed by the hAgo2 apo form. ClusPro (https://cluspro.org),^55^ Zdock(https://zlab.umassmed.edu/zdock/),^56^ and PatchDock (https://bioinfo3d.cs.tau.ac.il/PatchDocK/),^57^ were used as protein–protein docking algorithms to model the interaction in hAgo2-Hsp90. The three docking softwares predict protein–protein interactions by means of rigid-body docking using the structural information derived from the two interacting partners individually. No prior information about the binding site and/or additional energy options were added in our search. Different scoring functions are implemented in each of the docking algorithm to rank low-energy docked complexes. Docking predictions were performed either constraining or not the interaction at the N/M domains of the chaperone, based on literature data.^9,15,16^ A total of 180 complexes were generated, filtered according to the software scoring function, and therefore subjected to careful structural analyses and considerations. In particular, a cluster analysis on backbone atoms (cutoff: rmsd 3 nm) was applied to the full set of predicted complexes and a visual inspection guided the screening of the most representative complexes. Significantly, from a statistical perspective, 29% of the 180 docked complexes presented a preferred interaction surface localized at the N-domain level of the Argonaute protein, in line with experimental evidence.^8^ In the end, four hAgo2-Hsp90 complexes were selected for molecular dynamics simulations as reported above.

### Collective Motion Analysis

To extract functionally relevant movements, a principal component analysis (PCA) has been applied to MD simulations in order to filter global, collective motions from local, fast motions.^58^ The concerted motions associated with the largest collective atomic fluctuations (i.e., that account for the largest contribution to the atomic root mean deviations) are recovered by the principal eigenvectors (essential modes) of the covariance matrix of the given dynamic ensemble. PCA analysis was carried out on Cα atoms of hAgo2 protein along the meta-trajectory. A total of 5000 snapshots per trajectory per system are projected on the essential subspace described by the first two eigenvectors responsible for the maximum variation in conformation observed along molecular simulation. hAgo2 simulation in its apo state is used as reference and the conformational space sampled by hAgo2 bound to Hsp90 in the four complexes is projected onto apo-hAgo2 essential subspace. Therefore, the comparison among the conformational space spanned by hAgo2 in the apo state and in different interaction complexes can be evaluated. The two first eigenvectors account for the 55% and 12% of the total variance of the simulation.

### Energy Decomposition Method

The energy decomposition method (EDM)^37,38^ yields an interaction matrix **M***_ij_*, obtained by averaging the interaction energies between residue pairs, comprising all the nonbonded inter-residue atomic energy components (namely, van der Waals and electrostatic), calculated over the structures visited during a MD trajectory or the representative conformation of the most populated cluster. The method builds on a simplified picture of the most relevant residue–residue interactions in a certain fold. For a protein of *N* residues, this calculation produces an *N* × *N* matrix. The matrix **M***_ij_* can be diagonalized and re-expressed in terms of eigenvalues and eigenvectors, in the form
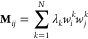
where *N* is the number of amino acids in the protein, *λ*_*k*_ is an eigenvalue, and *w*_*i*_^*k*^ is the *i*th component of the associated normalized eigenvector. The matrix of the pair energy-couplings corresponding to the first eigenvector is chosen to filter the contact map and identify all inter-residues low-energy contributions.

### Distance Fluctuation Analysis

Distance fluctuation DF_*ji*_ is defined as the time-dependent mean square fluctuation of the distance **r**_**i**j_ between Cα atoms of residues i and j:
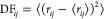
where brackets indicate the time-average over the trajectory. Low DF values indicate highly coordinated residues.^32^

### Center of Mass (COM) Distances Analysis

Distance length evolution between the two hAgo2 subdomains centroids were analyzed by the VMD tools package.^52^ Centroids were defined as the center of mass of PIWI (amino acids 496–797) and PAZ domains (amino acids 231–365). The distance between the two obtained geometric centers was calculated along simulation time for complexes **2** and **4**.

## Supporting Information Available

Figure S1, 3D structure of h human argonaute bound to target RNA; Figure S2, selected docking poses for hAgo2-Hsp90 complexes in different orientations; Figure S3, statistical distribution of the rotational angle described by PIWI and PAZ domains along the simulation time; Figure S4, nonbonded pair-interactions energies of hAgo2 in the apo form (at 300 K) and in complex **4**; Figure S5, distance fluctuations analysis of the hAgo2 in the apo form (at 300 K) and in complex **4**; Figure S6, extreme projections on principal modes derived from ED analysis in the unbound protein at 300 K and in complex **4**; Figure S7, effect of mutations on protein stability; Figure S8, structural comparison among Hsp90-client complexes; and Table S1, changes in protein stability upon mutations (PDF)
Coordinates of hAgo2-Hsp90_1 (PDB)
Coordinates of hAgo2-Hsp90_2 (PDB)
Coordinates of hAgo2-Hsp90_3 (PDB)
Coordinates of hAgo2-Hsp90_4 (PDB)

## Supplementary Material

ci0c00053_si_001.pdf

ci0c00053_si_002.pdb

ci0c00053_si_003.pdb

ci0c00053_si_004.pdb

ci0c00053_si_005.pdb

## References

[ref1] Liu J.; Carmell M. A.; Rivas F. V.; Marsden C. G.; Thomson J. M.; Song J.; Hammond S. M.; Joshua-Tor L.; Hannon G. J. Argonaute2 is the catalytic engine of mammalian RNAi. Science 2004, 305, 1437–1441. 10.1126/science.1102513. 15284456

[ref2] Meister G.; Landthaler M.; Patkaniowska A.; Dorsett Y.; Teng G.; Tuschl T. Human Argonaute2 mediates RNA cleavage targeted by miRNAs and siRNAs. Mol. Cell 2004, 15, 185–197. 10.1016/j.molcel.2004.07.007. 15260970

[ref3] Sheu-Gruttadauria J.; MacRae I. J. Structural Foundations of RNA Silencing by Argonaute. J. Mol. Biol. 2017, 429, 2619–2639. 10.1016/j.jmb.2017.07.018. 28757069 PMC5576611

[ref4] Kwak P. B.; Tomari Y. The N domain of Argonaute drives duplex unwinding during RISC assembly. Nat. Struct. Mol. Biol. 2012, 19, 145–151. 10.1038/nsmb.2232. 22233755

[ref5] Wang Y.; Juranek S.; Sheng G.; Li H.; Tuschl T.; Patel D. J. Structure of an argonaute silencing complex with a seed-containing guide DNA and target RNA duplex. Nature 2008, 456, 921–926. 10.1038/nature07666. 19092929 PMC2765400

[ref6] Wang Y.; Sheng G.; Juranek S.; Tuschl T.; Patel D. J. Structure of the guide-strand-containing argonaute silencing complex. Nature 2008, 456, 209–213. 10.1038/nature07315. 18754009 PMC4689319

[ref7] Wang Y.; Juranek S.; Li H.; Sheng G.; Wardle G. S.; Tuschl T.; Patel D. J. Nucleation, propagation and cleavage of target RNAs in Ago silencing complexes. Nature 2009, 461, 754–761. 10.1038/nature08434. 19812667 PMC2880917

[ref8] Kwak P. B.; Tomari Y. The N domain of Argonaute drives duplex unwinding during RISC assembly. Nat. Struct. Mol. Biol. 2012, 19, 145–151. 10.1038/nsmb.2232. 22233755

[ref9] Iwasaki S.; Kobayashi M.; Yoda M.; Sakaguchi Y.; Katsuma S.; Suzuki T.; Tomari Y. Hsc70/Hsp90 chaperone machinery mediates ATP-dependent RISC loading of small RNA duplexes. Mol. Cell 2010, 39, 292–299. 10.1016/j.molcel.2010.05.015. 20605501

[ref10] Johnston M.; Geoffroy M.-C.; Sobala A.; Hay R.; Hutvagner G. HSP90 Protein Stabilizes Unloaded Argonaute Complexes and Microscopic P-bodies in Human Cells. Mol. Biol. Cell 2010, 21, 1462–1469. 10.1091/mbc.e09-10-0885. 20237157 PMC2861606

[ref11] Miyoshi T.; Takeuchi A.; Siomi H.; Siomi M. C. A direct role for Hsp90 in pre-RISC formation in Drosophila (Nature Structural and Molecular Biology (2010) 17 (1024–1026)). Nat. Struct. Mol. Biol. 2011, 18, 516 10.1038/nsmb0411-516a. 20639883

[ref12] Iwasaki S.; Sasaki H. M.; Sakaguchi Y.; Suzuki T.; Tadakuma H.; Tomari Y. Defining fundamental steps in the assembly of the Drosophila RNAi enzyme complex. Nature 2015, 521, 533–536. 10.1038/nature14254. 25822791

[ref13] Guo Y.; Liu J.; Elfenbein S. J.; Ma Y.; Zhong M.; Qiu C.; Ding Y.; Lu J. Characterization of the mammalian miRNA turnover landscape. Nucleic Acids Res. 2015, 43, 2326–2341. 10.1093/nar/gkv057. 25653157 PMC4344502

[ref14] Nakanishi K.; Weinberg D. E.; Bartel D. P.; Patel D. J. Structure of yeast Argonaute with guide RNA. Nature 2012, 486, 368–374. 10.1038/nature11211. 22722195 PMC3853139

[ref15] Nakanishi K. Anatomy of RISC: how do small RNAs and chaperones activate Argonaute proteins?. Wiley Interdisciplinary Reviews: RNA 2016, 7, 637–660. 10.1002/wrna.1356. 27184117 PMC5084781

[ref16] Yoda M.; Kawamata T.; Paroo Z.; Ye X.; Iwasaki S.; Liu Q.; Tomari Y. ATP-dependent human RISC assembly pathways. Nat. Struct. Mol. Biol. 2010, 17, 17–24. 10.1038/nsmb.1733. 19966796 PMC2915567

[ref17] Elkayam E.; Kuhn C. D.; Tocilj A.; Haase A. D.; Greene E. M.; Hannon G. J.; Joshua-Tor L. The structure of human argonaute-2 in complex with miR-20a. Cell 2012, 150, 100–110. 10.1016/j.cell.2012.05.017. 22682761 PMC3464090

[ref18] Tsuboyama K.; Tadakuma H.; Tomari Y. Conformational Activation of Argonaute by Distinct yet Coordinated Actions of the Hsp70 and Hsp90 Chaperone Systems. Mol. Cell 2018, 70, 722–729. 10.1016/j.molcel.2018.04.010. 29775584

[ref19] Pearl L. H.; Prodromou C. Structure and Mechanism of the Hsp90 Molecular Chaperone Machinery. Annu. Rev. Biochem. 2006, 75, 271–294. 10.1146/annurev.biochem.75.103004.142738. 16756493

[ref20] Taipale M.; Krykbaeva I.; Koeva M.; Kayatekin C.; Westover K. D.; Karras G. I.; Lindquist S. Quantitative analysis of Hsp90-client interactions reveals principles of substrate recognition. Cell 2012, 150, 987–1001. 10.1016/j.cell.2012.06.047. 22939624 PMC3894786

[ref21] Paladino A.; Marchetti F.; Ponzoni L.; Colombo G. The Interplay between Structural Stability and Plasticity Determines Mutation Profiles and Chaperone Dependence in Protein Kinases. J. Chem. Theory Comput. 2018, 14, 1059–1070. 10.1021/acs.jctc.7b00997. 29262682

[ref22] Nykänen A.; Haley B.; Zamore P. D. ATP requirements and small interfering RNA structure in the RNA interference pathway. Cell 2001, 107, 309–321. 10.1016/S0092-8674(01)00547-5. 11701122

[ref23] Saibil H. Chaperone machines for protein folding, unfolding and disaggregation. Nat. Rev. Mol. Cell Biol. 2013, 14, 630–642. 10.1038/nrm3658. 24026055 PMC4340576

[ref24] Naruse K.; Matsuura-Suzuki E.; Watanabe M.; Iwasaki S.; Tomari Y. In vitro reconstitution of chaperone-mediated human RISC assembly. RNA 2018, 24, 6–11. 10.1261/rna.063891.117. 28971854 PMC5733571

[ref25] Yao C.; Sasaki H. M.; Ueda T.; Tomari Y.; Tadakuma H. Single-Molecule Analysis of the Target Cleavage Reaction by the Drosophila RNAi Enzyme Complex. Mol. Cell 2015, 59, 125–132. 10.1016/j.molcel.2015.05.015. 26140368

[ref26] Yuan Y. R.; Pei Y.; Ma J.; Kuryavyi V.; Zhadina M.; Meister G.; Chen H.; Dauter Z.; Tuschl T.; Patel D. J. Crystal structure of A. aeolicus argonaute, a site-specific DNA-guided endoribonuclease, provides insights into RISC-mediated mRNA cleavage. Mol. Cell 2005, 19, 405–419. 10.1016/j.molcel.2005.07.011. 16061186 PMC4689305

[ref27] Wang Y.; Juranek S.; Sheng G.; Li H.; Tuschl T.; Patel D. J. Structure of an argonaute silencing complex with a seed-containing guide DNA and target RNA duplex. Nature 2008, 456, 921–926. 10.1038/nature07666. 19092929 PMC2765400

[ref28] Schirle N. T.; MacRae I. J. The crystal structure of human argonaute2. Science 2012, 336, 1037–1040. 10.1126/science.1221551. 22539551 PMC3521581

[ref29] Schirle N. T.; Sheu-Gruttadauria J.; MacRae I. J. Structural basis for microRNA targeting. Science 2014, 346, 608–613. 10.1126/science.1258040. 25359968 PMC4313529

[ref30] Schirle N. T.; Sheu-Gruttadauria J.; Chandradoss S. D.; Joo C.; MacRae I. J. Water-mediated recognition of t1-adenosine anchors Argonaute2 to microRNA targets. eLife 2015, 4, 07646 10.7554/eLife.07646. PMC4606517 26359634

[ref31] Frank F.; Sonenberg N.; Nagar B. Structural basis for 5′-nucleotide base-specific recognition of guide RNA by human AGO2. Nature 2010, 465, 818–822. 10.1038/nature09039. 20505670

[ref32] Morra G.; Potestio R.; Micheletti C.; Colombo G. Corresponding functional dynamics across the Hsp90 chaperone family: Insights from a multiscale analysis of MD simulations. PLoS Comput. Biol. 2012, 8, e1002433 10.1371/journal.pcbi.1002433. 22457611 PMC3310708

[ref33] Ferraro M.; D’Annessa I.; Moroni E.; Morra G.; Paladino A.; Rinaldi S.; Compostella F.; Colombo G. Allosteric modulators of Hsp90 and Hsp70: Dynamics meets Function through Structure-Based Drug Design. J. Med. Chem. 2019, 62, 60–87. 10.1021/acs.jmedchem.8b00825. 30048133

[ref34] Roe S. M.; Ali M. M. U.; Meyer P.; Vaughan C. K.; Panaretou B.; Piper P. W.; Prodromou C.; Pearl L. H. The Mechanism of Hsp90 Regulation by the Protein Kinase-Specific Cochaperone p50cdc37. Cell 2004, 116, 87–98. 10.1016/S0092-8674(03)01027-4. 14718169

[ref35] D’ Annessa I. D.; Raniolo S.; Limongelli V.; Di Marino D.; Colombo G.; et al. J. Chem. Theory Comput. 2019, 15, 6368–6381. 10.1021/acs.jctc.9b00319. 31538783

[ref36] Daura X.; Jaun B.; Seebach D.; van Gunsteren W. F.; Mark A. E. Reversible peptide folding in solution by molecular dynamics simulation. J. Mol. Biol. 1998, 280, 925–32. 10.1006/jmbi.1998.1885. 9671560

[ref37] Genoni A.; Morra G.; Colombo G. Identification of domains in protein structures from the analysis of intramolecular interactions. J. Phys. Chem. B 2012, 116, 3331–3343. 10.1021/jp210568a. 22384792

[ref38] Tiana G.; Simona F.; De Mori G. M. S.; Broglia R. A.; Colombo G. Understanding the determinants of stability and folding of small globular proteins from their energetics. Protein Sci. 2004, 13, 113–124. 10.1110/ps.03223804. 14691227 PMC2286534

[ref39] Capriotti E.; Fariselli P.; Casadio R. I-Mutant2.0: Predicting stability changes upon mutation from the protein sequence or structure. Nucleic Acids Res. 2005, 33, W306–W310. 10.1093/nar/gki375. 15980478 PMC1160136

[ref40] Pandurangan A. P.; Ochoa-Montaño B.; Ascher D. B.; Blundell T. L. SDM: A server for predicting effects of mutations on protein stability. Nucleic Acids Res. 2017, 45, W229–W235. 10.1093/nar/gkx439. 28525590 PMC5793720

[ref41] Kuzmanic A.; Pritchard R. B.; Hansen D. F.; Gervasio F. L. Importance of the Force Field Choice in Capturing Functionally Relevant Dynamics in the von Willebrand Factor. J. Phys. Chem. Lett. 2019, 10, 1928–1934. 10.1021/acs.jpclett.9b00517. 30933516 PMC6475856

[ref42] Vaughan C. K.; Göhlke U.; Sobott F.; Good V. M.; Ali M.M. U.; Prodromou C.; Robinson C. V.; Saibil H. R.; Pearl L. H. Structure of an Hsp90-Cdc37-Cdk4 Complex. Mol. Cell 2006, 23, 697–707. 10.1016/j.molcel.2006.07.016. 16949366 PMC5704897

[ref43] Verba K. A.; Wang R. Y.; Arakawa A.; Liu Y.; Shirouzu M.; Yokoyama S.; Agard D. A. Atomic structure of Hsp90:Cdc37:Cdk4 reveals Hsp90 regulates kinase via dramatic unfolding. Science 2016, 352, 1542 10.1126/science.aaf5023. 27339980 PMC5373496

[ref44] Taipale M.; Krykbaeva I.; Koeva M.; Kayatekin C.; Westover K. D.; Karras G. I.; Lindquist S. Quantitative analysis of Hsp90-client interactions reveals principles of substrate recognition. Cell 2012, 150, 987–1001. 10.1016/j.cell.2012.06.047. 22939624 PMC3894786

[ref45] Taipale M.; Krykbaeva I.; Whitesell L.; Santagata S.; Zhang J.; Liu Q.; Gray N. S.; Lindquist S. Chaperones as thermodynamic sensors of drug-target interactions reveal kinase inhibitor specificities in living cells. Nat. Biotechnol. 2013, 31, 630–637. 10.1038/nbt.2620. 23811600 PMC3774174

[ref46] Jiang H.; Sheong F. K.; Zhu L.; Gao X.; Bernauer J.; Huang X. Markov State Models Reveal a Two-Step Mechanism of miRNA Loading into the Human Argonaute Protein: Selective Binding followed by Structural Re-arrangement. PLoS Comput. Biol. 2015, 11, e1004404 10.1371/journal.pcbi.1004404. 26181723 PMC4504477

[ref47] Case D. A.; Darden T. A.; Cheatham T. E.; Simmerling C. L.; Wang J.; Duke R. E.; Luo G.; Walker R. C.; Zhang W.; Merz K. M.; Roberts B.; Hayik S.; Roitberg A.; Seabra G.; Swails J.; Götz A. W.; Kolossváry I.; Wong K. F.; Paesani F.; Vanicek J.; Wolf R. M.; Liu J.; Wu X.; Brozell S. R.; Steinbrecher T.; Gohlke H.; Cai Q.; Ye X.; Wang J.; Hsieh M. J.; Cui G.; Roe D. R.; Mathews D. H.; Seetin M. G.; Salomon-Ferrer R.; Sagui C.; Babin V.; Luchko T.; Gusarov S.; Kovalenko A.; Kollman P. A. Amber 12; University of California, San Francisco: 2012.

[ref48] Lindorff-Larsen K.; Piana S.; Palmo K.; Maragakis P.; Klepeis J. L.; Dror R. O.; Shaw D. E. Improved side-chain torsion potentials for the Amber ff99SB protein force field. *Proteins Struct*. Proteins: Struct., Funct., Genet. 2010, 78, 1950–1958. 10.1002/prot.22711. 20408171 PMC2970904

[ref49] Jorgensen W. L.; Chandrasekhar J.; Madura J. D.; Impey R. W.; Klein M. L. Comparison of simple potential functions for simulating liquid water. J. Chem. Phys. 1983, 79, 926 10.1063/1.445869.

[ref50] Darden T.; York D.; Pedersen L. Particle mesh Ewald: An W log(N) method for Ewald sums in large systems. J. Chem. Phys. 1993, 98, 10089–10092. 10.1063/1.464397.

[ref51] Ryckaert J. P.; Ciccotti G.; Berendsen H. J. C. Numerical integration of the cartesian equations of motion of a system with constraints: molecular dynamics of n-alkanes. J. Comput. Phys. 1977, 23, 327–341. 10.1016/0021-9991(77)90098-5.

[ref52] Humphrey W.; Dalke A.; Schulten K. VMD: Visual molecular dynamics. J. Mol. Graphics 1996, 14, 33–38. 10.1016/0263-7855(96)00018-5. 8744570

[ref53] L DeLano W. The PyMOL Molecular Graphics System; DeLano Scientific: Palo Alto, CA; http://www.pymol.org. 2002.

[ref54] Pettersen E. F.; Goddard T. D.; Huang C. C.; Couch G.; Greenblatt D. M.; Meng E. C.; et al. UCSF Chimera - A visualization system for exploratory research and analysis. J. Comput. Chem. 2004, 25, 1605–1612. 10.1002/jcc.20084. 15264254

[ref55] Kozakov D.; Hall D. R.; Xia B.; Porter K. A.; Padhorny D.; Yueh C.; Beglov D.; Vajda S. The ClusPro web server for protein-protein docking. Nat. Protoc. 2017, 12, 255–278. 10.1038/nprot.2016.169. 28079879 PMC5540229

[ref56] Pierce B. G.; Wiehe K.; Hwang H.; Kim B. H.; Vreven T.; Weng Z. ZDOCK server: Interactive docking prediction of protein-protein complexes and symmetric multimers. Bioinformatics 2014, 30, 1771–1773. 10.1093/bioinformatics/btu097. 24532726 PMC4058926

[ref57] Schneidman-Duhovny D.; Inbar Y.; Nussinov R.; Wolfson H. J. PatchDock and SymmDock: Servers for rigid and symmetric docking. Nucleic Acids Res. 2005, 33, W363–W367. 10.1093/nar/gki481. 15980490 PMC1160241

[ref58] Amadei A.; Linssen A. B. M.; Berendsen H. J. C. Essential dynamics of proteins. Proteins: Struct., Funct., Genet. 1993, 17, 412–425. 10.1002/prot.340170408. 8108382

