# Supplementary material for: Mechanistic Model for the Hsp90-Driven Opening of Human Argonaute
Source: J Chem Inf Model. 2020 Feb 25;60(3):1469–80. doi: 10.1021/acs.jcim.0c00053 (PMC7997374; doi:10.1021/acs.jcim.0c00053)
Supplement: Supplementary file 1 — ci0c00053_si_001.pdf [file ci0c00053_si_001.pdf]

Supporting Information

Mechanistic Model for the Hsp90-Driven Opening of Human Argonaute

Silvia Rinaldi ,[1†] Giorgio Colombo ,[*1†,2‡] and Antonella Paladino [*1†,3§]

[1†]Istituto di Science e Tecnologie Chimiche “Giulio Natta” SCITEC, CNR, via Mario Bianco 9, 20131, Milan, Italy

[2‡]Dipartimento di Chimica, Università degli Studi di Pavia, Viale Taramelli 12, 27100 Pavia, Italy

[3§]BIOGEM Istituto di Ricerche Genetiche “G. Salvatore”, via Camporeale, 83031 Ariano Irpino, Italy

*Email: g.colombo@unipv.it

*Email: antonella.paladino@biogem.it

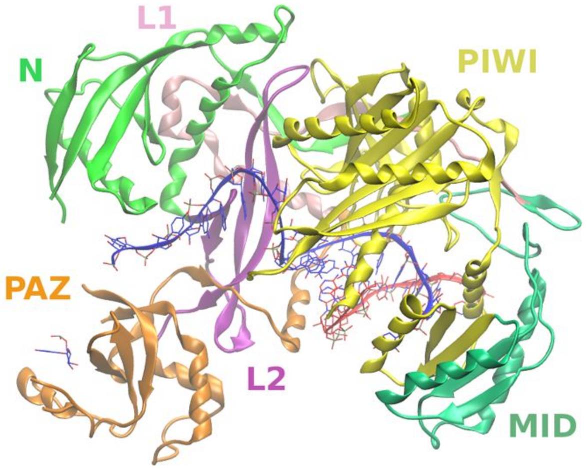

Figure S1. Human Argonaute Bound to Target RNA. 3D structure of hAgo2 is shown in cartoons: N-terminal, PAZ, MID and PIWI, connected by two linker domains (L1 and L2) are drawn with the same color codes as in Figure 1 and labelled. t1-C target and guide RNAs are indicated in red and blue ribbons, respectively. In hAgo2-RISC, the 5' monophosphate and base moiety are recognized at the cleft between MID and PIWI domains, while the 3' end is fastened at the PAZ domain. X-ray structure is withdrawn from RCSB with PDB access number 4Z4C.

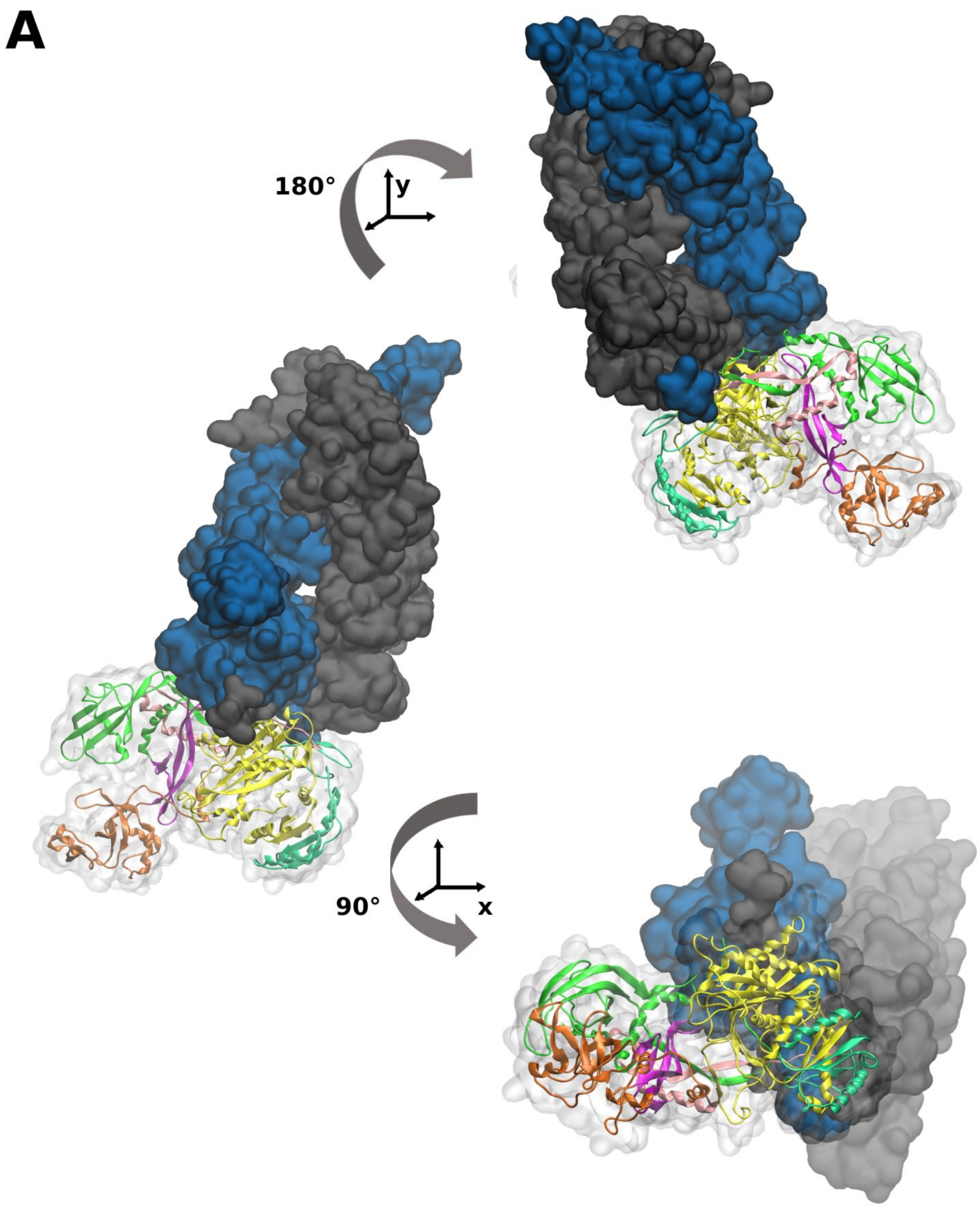
A
180°
y
90°
x

B

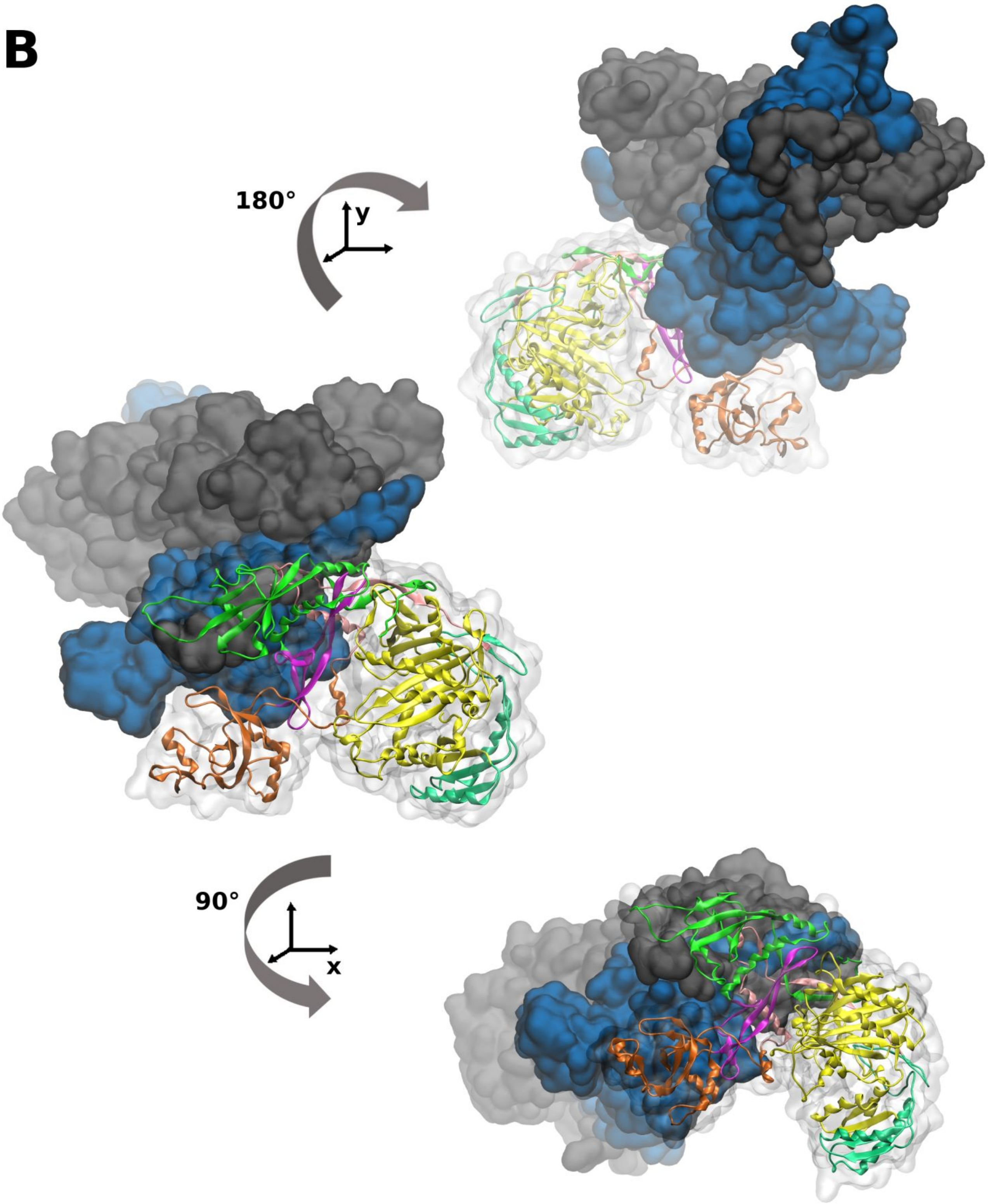

C

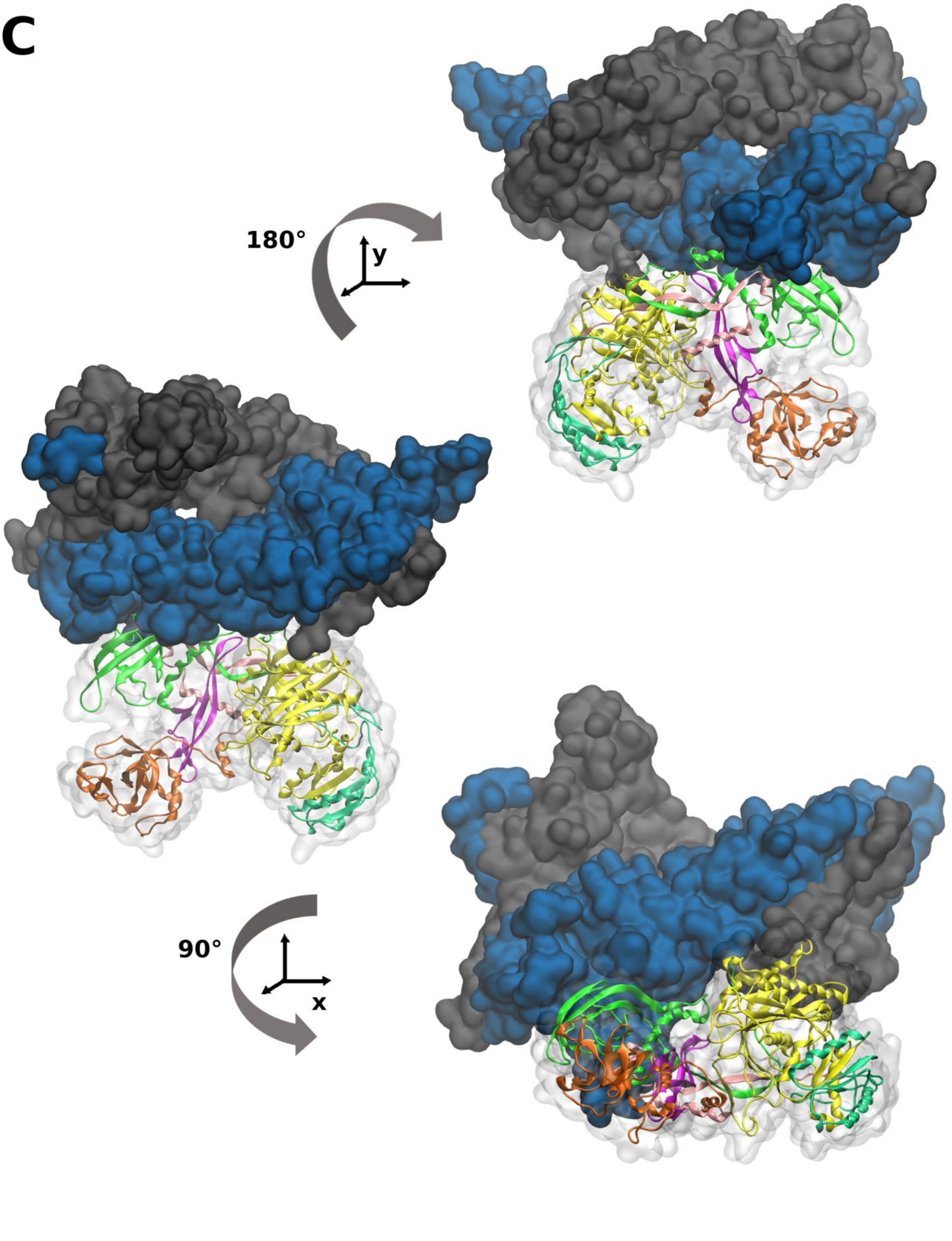

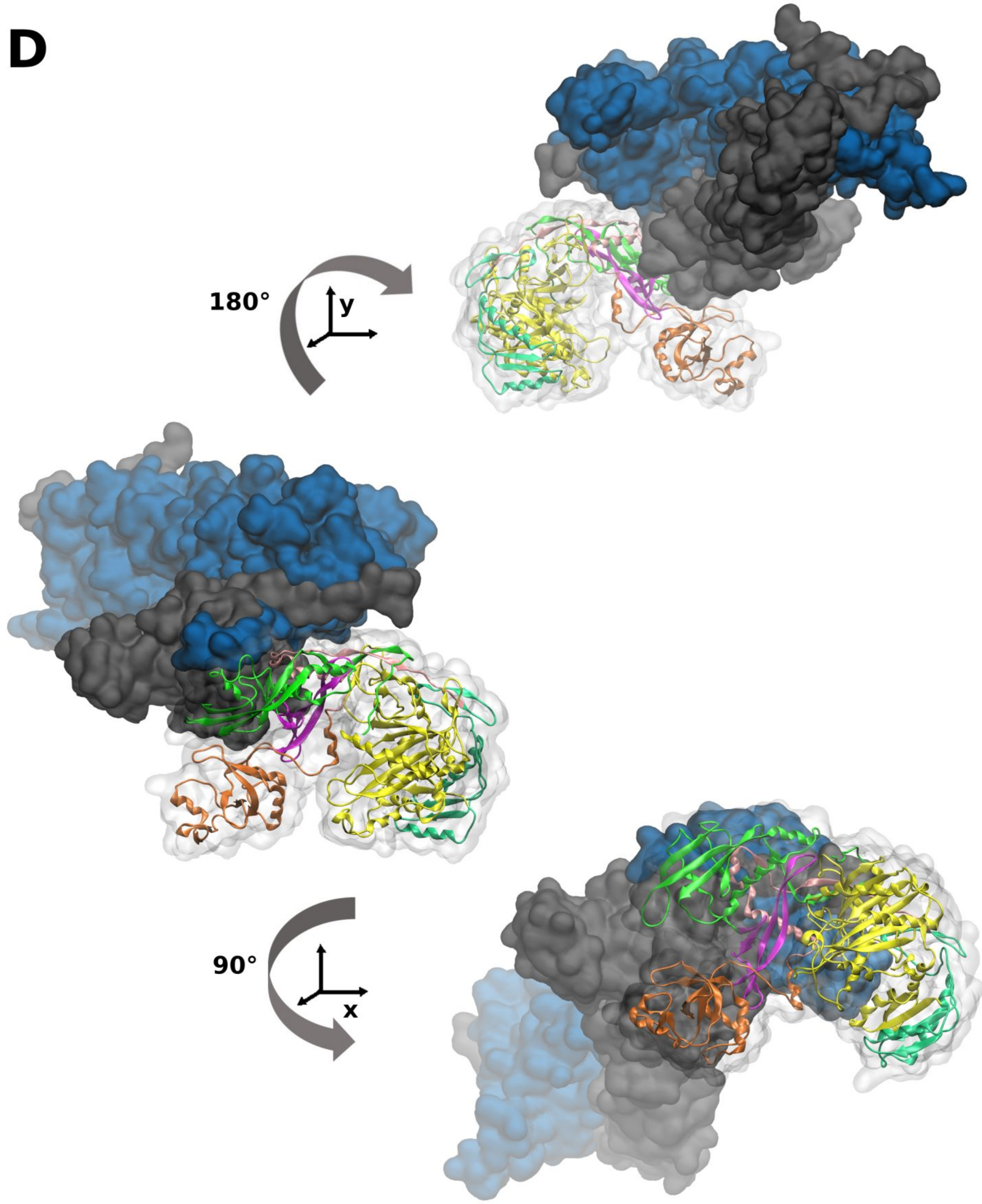

Figure S2. hAgo2-Hsp90 complexes. Selected docking poses for hAgo2-Hsp90 interaction (as in Figure 1). Different orientations are displayed in A (complex **1**), B (complex **2**), C (complex **3**) and D (complex **4**) after applying a 180° y-axis and a 90° x-axis rotation. hAgo2 is shown in cartoons (N-> green, PAZ -> orange, PIWI -> yellow, MID-> lime, L1 -> purple, L2 ->pink) and ghost surface. Blue and grey solid surface representation is used for Hsp90 subunits.

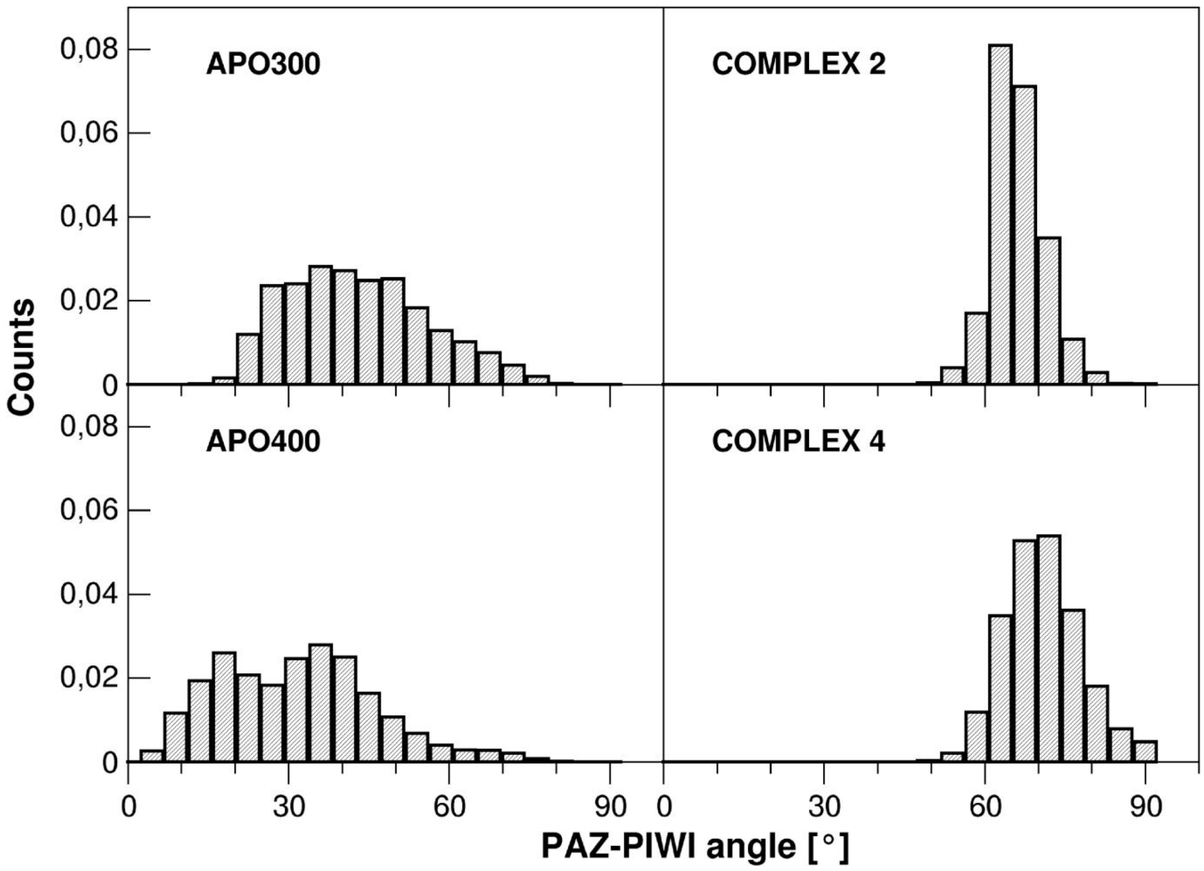

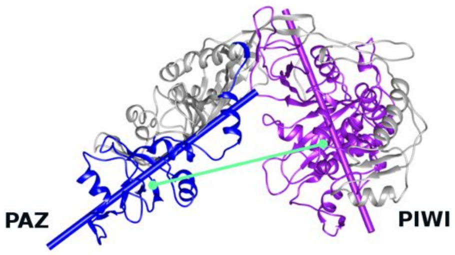

Figure S3. **Statistical distribution of the rotational angle described by PIWI and PAZ domains along the simulation time.** Histogram counts are normalized to 1 for the apo form of hAgo2 at 300 K, 400 K and in complex with Hsp90 (complexes **2** and **4**). Rotational angles are defined as the angle made by the two inertia axes passing through PAZ and PIWI domains, as indicated in the figure at the bottom.

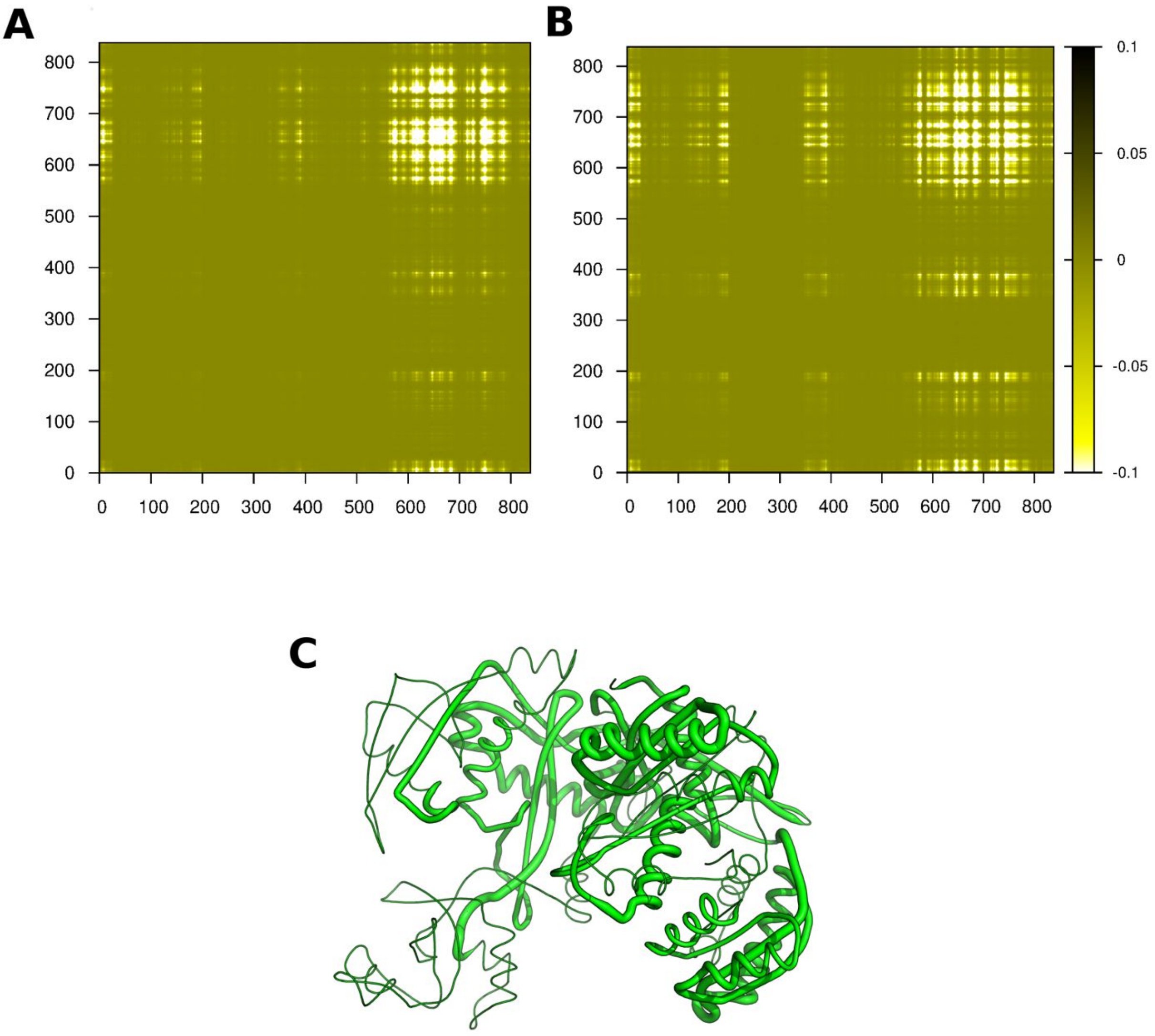

Figure S4. **EDM matrices**. Non-bonded pair-interactions energies are calculated on the most representative structure on hAgo2 in the apo form (300K) (A) and in complex 4 (taken as reference) (B). The regions of the protein that are stabilizing cores upon complex formation are highlighted in tube representation (C).

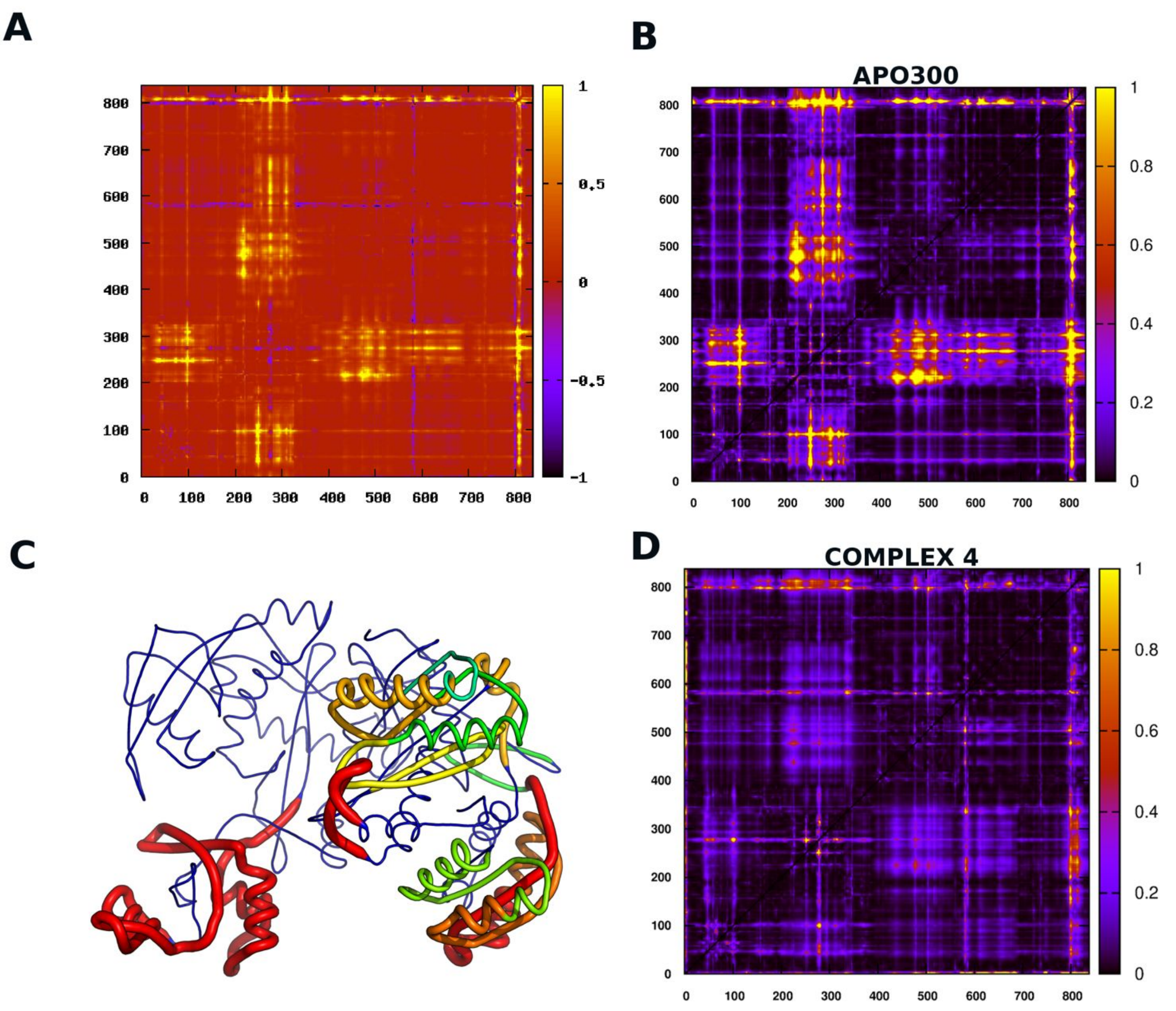

Figure S5. **DF matrices**. Distance Fluctuations of the hAgo2 in the apo form (300K) (B) and in complex 4 (taken as reference) (D). Difference matrix between the unbound and complexed Ago DF matrices (A); yellow spots indicate regions where Ago flexibility is higher in the unbound state. The regions of the protein where intrinsic flexibility is decreased upon complex formation are highlighted in tube representation (C).

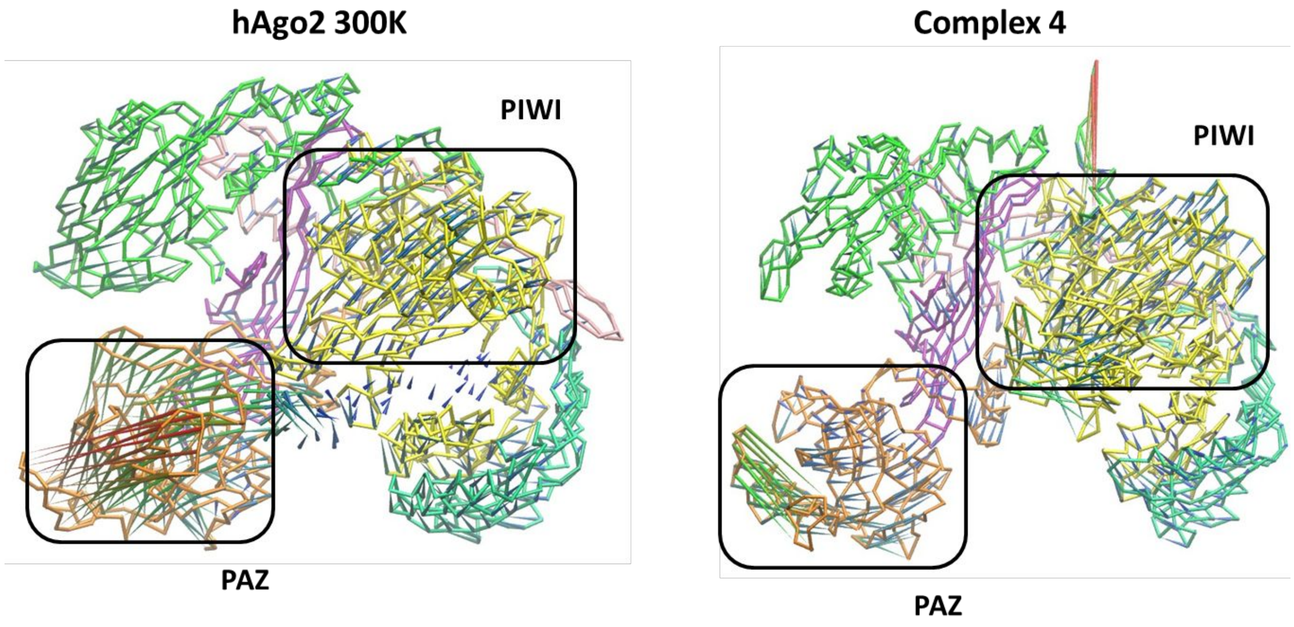

Figure S6. **Extreme projections on principal modes.** First eigenvector resulted from the PCA performed on hAgo2 Cα atoms in the unbound protein at 300 K (left) and in complex 4 (right).

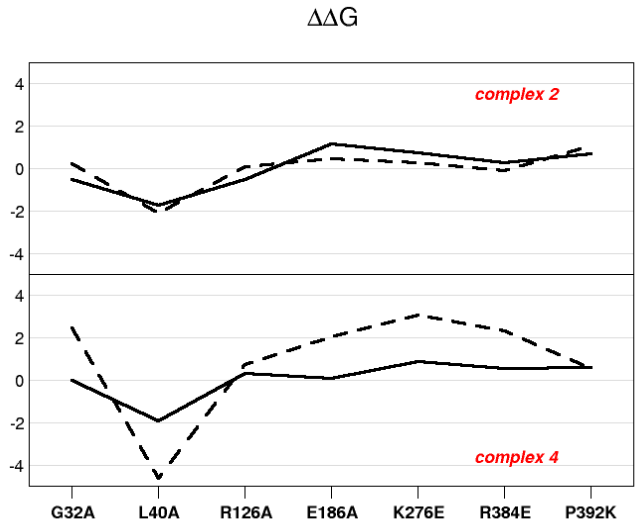

Figure S7. **Effect of mutations on protein stability.** Normalized ΔΔG data (given in kcal/mol) on y-axis are plotted for complexes 2 and 4. Solid line indicates results for I-Mutant, dashed line for SDM.

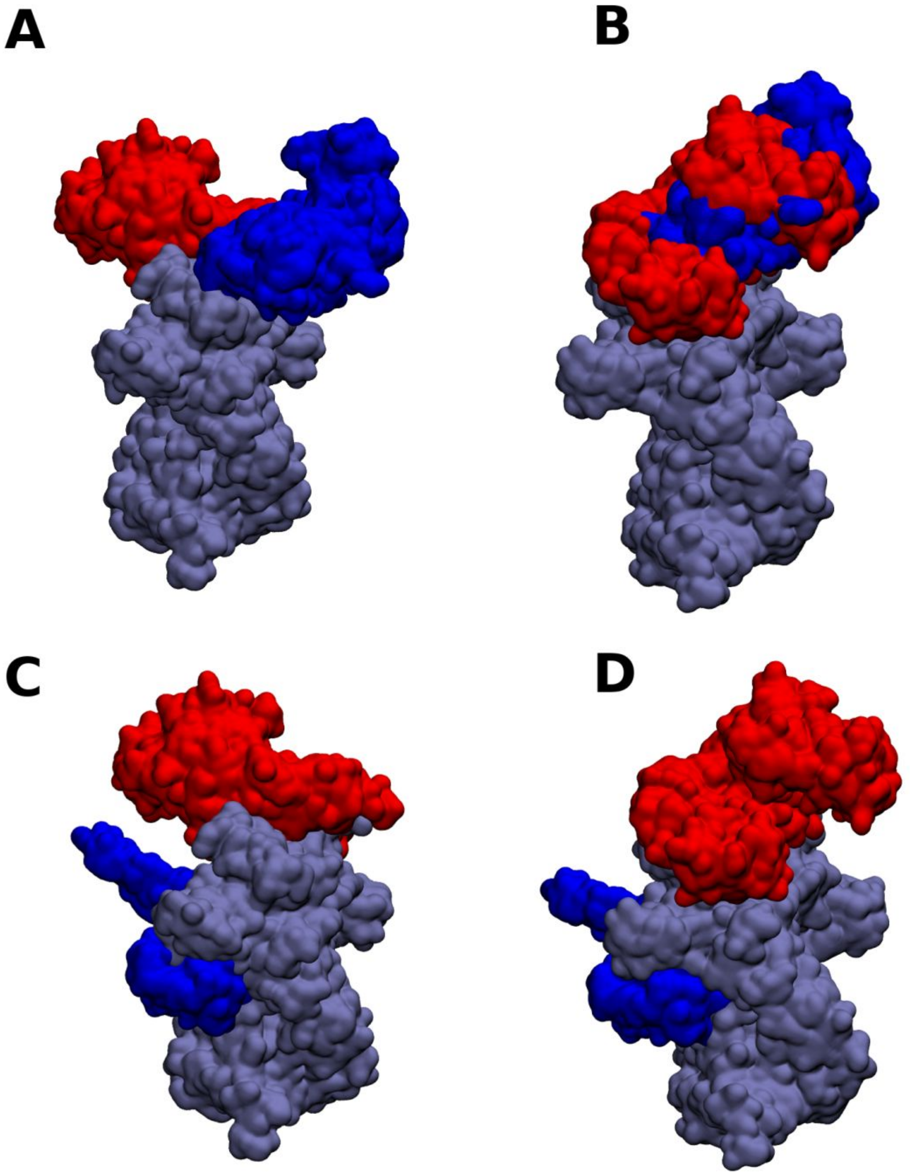

Figure S8. Structural comparison among Hsp90-client complexes. Superposition of complex 2 and 4 with 2JKI.pdb (A, B) and with 5FWK.pdb (C, D) respectively. Grey and red solid surface is used to represent Hsp90 and hAgo2 proteins. Blue solid surface is used to indicate Sgt1 (A,B) and Cdc37-Cdk4 (C,D).

Table S1. **Changes in protein stability upon mutations.** Prediction of the effects of single point mutations on Complex **2** and **4** are reported per I-mutant and SDM servers. Predicted ΔΔG are given in kcal/mol.*

mutation	Complex 2		Complex 4	
	I-mutant	SDM	I-mutant	SDM
G32A	-1,33	0,10	-1,12	0,5
L40A	-2,21	-3,97	-2,38	-3,92
R126A	-1,35	-0,14	-0,89	-0,59
E186A	-0,15	0,45	-1,04	0,22
K276E	-0,44	0,17	-0,53	0,87
R384E	-0,78	-0,47	-0,74	0,4
P392K	-0,48	1,54	-0,67	0,67

*ΔΔG > 0 indicate stabilizing mutations, ΔΔG < 0 indicate destabilizing mutations.
